# Stacked Nanosheet Gate‐All‐Around Morphotropic Phase Boundary Field‐Effect Transistors

**DOI:** 10.1002/advs.202413090

**Published:** 2025-03-17

**Authors:** Sihyun Kim, Hyun‐Min Kim, Ki‐Ryun Kwon, Daewoong Kwon

**Affiliations:** ^1^ Department of Electronic Engineering Sogang University Seoul 04107 Republic of Korea; ^2^ Department of Electrical Engineering Hanyang University Seoul 04763 Republic of Korea

**Keywords:** capacitance‐boosting, effective oxide thickness, field‐effect transistor, gate‐all‐around, high‐κ, hysteresis‐free, HZO, morphotropic phase boundary, nanosheet

## Abstract

A material design method is proposed using ferroelectric (FE)–antiferroelectric (AFE) mixed‐phase HfZrO_2_ (HZO) to achieve performance improvements in morphotropic phase boundary (MPB) field‐effect transistors (MPB‐FETs), such as steep subthreshold swing (*SS*) and non‐hysteretic on‐current (*I*_on_) enhancement. Capacitance (small‐signal and quasi‐static) and transient current measurements of MPB‐FETs confirmed that near‐threshold voltage (*V*_TH_) capacitance amplification leads to *I*_on_ boosts under high‐speed and low‐power conditions. For the first time, two‐stacked nanosheet (NS) gate‐all‐around (GAA) MPB‐FETs with optimized HZO, demonstrating superior short channel effect (SCE) immunity with enhanced current drivability is fabricated. Bias temperature instability (BTI) analyses revealed over‐10‐year endurance at 0.6 V and 120 °C. The NS MPB‐FETs achieved a 24.1% *I*_on_ gain, 82.5 mV operating voltage scalability, and a 30.7% AC performance improvement at *V*_DD_ = 0.6 V compared to control MOSFETs with HfO_2_ high‐k dielectric. Transconductance benchmarks with industrial logic technologies confirmed that the MPB with mixed HZO enables effective oxide thickness scaling without mobility degradation, making NS MPB‐FETs an ideal choice for low‐power / high‐performance CMOS technology.

## Graphical abstract

This study proposes a material design using ferroelectric‐antiferroelectric mixed‐phase HZO to achieve steep subthreshold swing and non‐hysteretic on‐current enhancement in morphotropic phase boundary field‐effect transistors (MPB‐FETs). For the first time, two‐stacked nanosheet GAA MPB‐FETs with optimized HZO are demonstrated, validating superior short‐channel effect immunity and enhanced current drivability, attributed to the scaled EOT enabled by the MPB thin film.

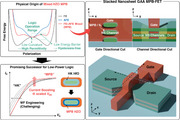

## 1 Introduction

The continuous miniaturization of metal‐oxide‐semiconductor field‐effect transistors (MOSFETs) is now being driven by gate‐all‐around (GAA) technology. Among various GAA configurations, vertically stacked horizontal nanosheet (NS) GAAFETs stand out as highly promising contenders for future complementary MOS (CMOS) technology, owing to their superior current drivability and exceptional gate control.^[^ ^1^, ^2^, ^3^, ^4^, ^5^ ^]^ Furthermore, unlike FinFETs, which offer only discrete tunability of current drivability, NS GAAFETs enable continuous current scaling within a chip through flexible nanosheet width (*W*_NS_) patterning. However, this flexibility introduces a critical challenge in extremely short‐channel devices: as *W*_NS_ increases to boost drivability, gate controllability degrades, exacerbating the short‐channel effect (SCE) and leading to a sharp rise in off‐state current (*I*_off_).^[^ ^6^ ^]^ To overcome this fundamental trade‐off and continue extending Moore's law through NS technology, a pivotal breakthrough is required. Further reducing the effective oxide thickness (EOT) to enhance gate controllability is the only viable path to achieving high drivability without compromising off‐state power density. EOT, which is the sum of the interfacial layer (IL) SiO_2_ thickness and the scaled high‐κ (HK) gate dielectric thickness (accounting for the permittivity ratio, ε_HK_/ε_IL_), faces stringent scaling limitations due to the IL. Shrinking the IL thickness is a formidable challenge, hindered by process complexities, reliability concerns, and mobility degradation.

**Figure 1:**
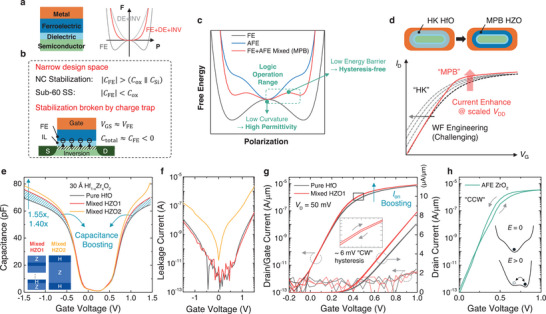
Material design for MPB‐FETs. a) Conventional NC stabilization strategy with the help of a series‐connected dielectric layer and corresponding free‐energy landscape. b) Crucial issues of NC stabilization using dielectric in MFIS NCFETs. c) Illustrated free‐energy landscape of FE, AFE, and FE‐AFE mixed phase (MPB) HZO films. d) Potential advantages of MPB‐FET over HK MOSFET: higher performance within scaled *V*_DD_ without challenging WF engineering and *I*_off_ increase. e,f) *C*–*V* and *I*–*V* curves of fabricated MFIS capacitors with pure HfO_2_, mixed HZO 1 and 2 stacks having the same thickness (30 Å). g) Measured *I*_D_–/*I*_G_–*V*_G_ characteristics of planar MPB‐FET compared to the pure HfO FET. (inset) An almost hysteresis‐free nature is exhibited in the enlarged *I*_D_–*V*_G_ curve of the HZO device. h) Hysteretic *I*_D_–*V*_G_ curves of AFE (ZrO_2_) FET and (inset) free‐energy landscape illustrating the field‐induced nonpolar‐to‐polar switching.

Recently, there has been growing interest in the innovative negative capacitance (NC) phenomenon in ferroelectric (FE) materials, as it offers a breakthrough beyond the physical limitations of MOSFETs.^[^ ^7^, ^8^, ^9^, ^10^, ^11^, ^12^, ^13^ ^]^ The NC effect arises from the thermodynamic behavior of FE films, where an unstable region with a negative curvature in the Gibbs free energy landscape (d^2^ *E*_FE_/d*P* ^2^ ≈*C*_FE_^−1^ < 0, where *E*_FE_, *P*, and *C*_FE_ represent the FE energy, polarization, and capacitance, respectively) between the two stable energy wells indicates the NC nature under limited conditions.^[^ ^14^ ^]^ Notably, atomic layer deposition (ALD)‐based doped‐HfO_2_ has emerged as a highly promising FE material for state‐of‐the‐art CMOS devices due to its process compatibility and thickness scalability down to nanometer thicknesses.^[^ ^15^, ^16^, ^17^, ^18^ ^]^ Nonetheless, realizing NC in practical applications remains challenging due to its inherent thermodynamic instability, raising critical questions about the real‐world existence and reliability of NC. Several research groups have attempted to experimentally observe NC in ferroelectric materials.^[^ ^19^, ^20^, ^21^, ^22^, ^23^ ^]^ Michael Hoffmann et al. calculated a double‐well free‐energy landscape from the voltage and current measurements using a ferroelectric capacitor with an additional dielectric layer and a series‐connected resistor, which delayed charge screening during transient voltage pulses.^[^ ^22^ ^]^ This phenomenon, observed when charge screening by metal electrodes is slower than polarization switching in fast measurements, is referred to as the transient NC. Because transient NC involves polarization switching, it generates hysteresis in transfer characteristics and requires a significant amount of voltage or time.^[^ ^7^, ^8^, ^24^, ^25^ ^]^ As such, transient observations of NC do not conclusively establish its viability for logic devices. To address this, research has explored the thermodynamic stabilization of NC (energy landscape of **Figure** **1**a) using a series‐connected dielectric and inversion capacitances in a metal‐FE‐IL‐semiconductor (MFIS) stack. The NC can be stabilized under specific conditions (|*C*_FE_ | > (*C*_ox_ || *C*_Si_)).^[^ ^26^ ^]^ However, in advanced CMOS technologies that rely on high‐κ dielectric, an IL SiO_2_ is indispensable to reduce interface trap density (*D*_it_), and must be kept extremely thin (∼ several angstroms) for EOT scaling, which conflicts with the requirement for stable NC through a series‐connected dielectric.^[^ ^27^ ^]^ Moreover, only a narrow design space satisfies the capacitance conditions necessary for achieving both stabilized NC and sub‐thermionic subthreshold swing (*SS*). However, even when the capacitance‐matching conditions are marginally met, the stabilized NC state is highly susceptible to disruption from charge screening via charge injection at the FE/IL interface (Figure 1b).

The recent discovery of FE‐AFE mixed‐phase morphotropic phase boundary (MPB) materials offers a promising solution.^[^ ^28^, ^29^ ^]^ These mixed‐phase MPB promote a capacitance‐boosting effect with negligible hysteresis, originating from the stabilized NC itself, without the help of a second dielectric, namely, an IL (Figure 1c). Capacitance boosting observed in MPB materials at a low electric field (E‐field) enables an increase in on‐current (*I*_on_) within the typical operating range of logic devices. Hence, MPB‐FETs are believed to offer higher performance without sacrificing *I*_off_ or requiring complex work‐function engineering, as typically employed in advanced HK technologies (Figure 1d), thereby allowing further scaling of the supply voltage (*V*_DD_). However, previous MPB‐FET studies^[^ ^28^, ^29^ ^]^ have only demonstrated the capacitance‐boosting effect on metal‐ferroelectric‐metal (MFM) or MFIS capacitors, without providing physical evidence of what occurs in the MPB thin film at the FET level, which must be clarified to optimize the materials for MPB‐FET fabrication

In this study, we investigated the capacitance‐boosting effect of mixed‐phase HfZrO_2_ (HZO) through MFM and MFIS capacitor experiments and verified the resulting current enhancement by applying the MPB stack to silicon‐on‐insulator (SOI) planar FETs. Especially, FET‐level verification of capacitance boosting was conducted through a small signal and quasi *C*–*V* approach. This work presents, for the first time, the experimental demonstration of 2‐stacked single crystalline Si NS GAA MPB‐FETs, validating their transfer characteristics, bias temperature instability (BTI), and AC performance. Our findings confirm the stabilized (hysteresis‐free) behavior of the NS GAA MPB‐FETs with superior performance, highlighting their potential for future low‐power/high‐performance CMOS technology.

## 2 Results and Discussion

### 2.1 Origin of Hysteresis‐Free Operation in FE‐AFE mixed phase HZO

In this study, an ultrathin HfO_2_‐ZrO_2_ superlattice gate stack exploiting the mixed FE–AFE order was stabilized at a 3‐nm thickness. The hysteresis‐free operations of the mixed‐phase MPB (Figure 1c) can be explained as follows. The origin of the antiferroelectricity in both ZrO_2_ and HfO_2_ was investigated by examining the field‐induced tetragonal‐to‐orthorhombic (nonpolar‐to‐polar) phase interconversion. Consequently, at low electric fields, the behavior of the mixed FE–AFE state resembles that of a FE–dielectric (polar–nonpolar) heterostructure. This similarity generates depolarization fields in the FE layer, aided by a series‐connected dielectric analogous to the effects observed in NC stabilization. The lateral coexistence of nonpolar and polar phases within mixed‐phase HZO plays a crucial role in facilitating the flattening of the ferroelectric energy landscape through the depolarization fields mentioned above. Additionally, previous studies have shown that structural inhomogeneities with heterogeneous elastic energies can lead to the destabilization of long‐range polarization, suppression of polarization, and consequent flattening of energy landscapes.^[^ ^19^ ^.^ ^30^, ^31^ ^]^ The introduction of in‐plane polarization devices increases the depolarization field due to the electrostatic coupling with the nonpolar AFE phase in the lateral direction. The utilization of inhomogeneity to generate depolarization fields and enhance the susceptibility has been successfully demonstrated in perovskites that exhibit heterogeneous polar–nonpolar regions, as indicated in previous studies.^[^ ^29^ ^]^ Building on these underlying mechanisms, our research illustrates the feasibility of stabilizing mixed nonpolar‐polar phase competition in 3‐nm‐thick binary oxide films, consequently augmenting their permittivity with negligible hysteresis. Notably, the flattening of the energy landscape through depolarization fields operates on the same fundamental principle, regardless of the charge injection at the FE/IL boundary, as the NC stabilization with the help of series‐connected dielectrics where depolarization fields locally stabilize the ferroelectric state at a higher energy level than the ground state of an isolated and homogeneous FE, resulting in energy landscapes with negative curvature.^[^ ^32^, ^33^ ^]^

### 2.2 MPB Material Optimization in MFM/MFIS Capacitors

The FE–AFE mixed phase (MPB) can be obtained by increasing the Zr content from 0.6 to 0.8 in the Hf_1‐_ *_x_* Zr*_x_* O_2_ thin film (*t*_HZO_ = 60 Å). We analyzed the capacitance and polarization properties of the fabricated MFM capacitors with various Zr ratios (*x* = 0.5, 0.75, and 1.0) and laminated structures (Figure S1a‐c, Supporting Information).^[^ ^33^ ^]^ The small‐signal capacitance–voltage (*C*–*V*), and triangular pulse charge–voltage (*Q‐*‐*V*) characteristics of the MFM capacitors are depicted in Supporting Information Figures S1d‐f, confirming that the phase of the HZO stack can be adjusted from FE (*x* = 0.5) to AFE (*x* = 1.0), depending on the Zr content. In HZO with a well‐designed mixed phase, namely the MPB (*x* = 0.75), the capacitance is significantly augmented in a smaller operating voltage range compared with FE and AFE. Table S1 (Supporting Information) provides benchmarking of our MPB material against previously reported MPB films.^[^ ^34^, ^35^, ^36^, ^37^ ^]^

Grazing‐angle incidence X‐ray diffraction (GIXRD) techniques indicate the presence of tetragonal and orthorhombic phases, corresponding to the AFE and FE orders in fluorite‐structured films, respectively. Figure S1g (Supporting Information) shows the GIXRD patterns of the FE, mixed‐phase (FE–AFE), and AFE films, which reveal distinct diffraction peaks between the tetragonal (t‐phase) and orthorhombic (o‐phase) phases. As shown in Figure S1h (Supporting Information), the ratio occupied by each phase was calculated after separating the three phases (Note S1, Supporting Information), and the phase ratios were obtained using the area ratios of the three o‐/t‐/m‐phase peaks.^[^ ^38^, ^39^ ^]^ Figure S1i (Supporting Information) compares the o‐/t‐/m‐phase ratios for the FE, mixed phase, and AFE, showing that the mixed‐phase HZO film exhibited a ferroelectric (33.6%)–antiferroelectric (65.1%) ratio while demonstrating the highest permittivity.

Subsequently, to verify the capacitance enhancement in the MFIS stack, which is identical to the gate stack of the MPB‐FET, capacitors with mixed HZO (*t*_HZO_ = 30 Å) were compared with those with pure HfO_2_ (*t*_HfO2_ = 30 Å). Both inversion and accumulation capacitances were measured, enabled by the self‐aligned n+ doping (Figure S2a–d, Supporting Information). In this experiment, two types of HZO films with distinct ALD stacking methods, solid solution (mixed HZO1) and nanolaminate structures (mixed HZO2), were employed (Figure S2e, Supporting Information). Figure 1e confirms the enhancement of the accumulation and inversion capacitances in the mixed HZO stacks 1 and 2 (40% and 55% gains over the pure HfO_2_ capacitor, respectively). Although mixed HZO2 exhibited higher gate capacitance, mixed HZO1 was selected as the MPB thin film due to the significant leakage current observed in Mixed HZO2 (Figure 1f). While the nano‐laminate structure has been reported as the best example of a mixed‐phase HZO with a significant capacitance enhancement,^[^ ^28^ ^]^ in our experiments, this structure resulted in unacceptable leakage current, likely due to ZrO_2_’s lower bandgap as well as the lower poly‐crystallization temperature compared to HfO_2_ or HZO solid solution. Transmission electron microscopy (TEM) and energy‐dispersive X‐ray spectroscopy (EDS) analyses of mixed HZO1 in Figure S2f (Supporting Information) confirmed that the ALD stack was composed of 9 Å of IL (SiO_2_) and 30 Å of crystallized HZO (*x* = 0.74), as intended.

### 2.3 Performance Enhancement of Planar MPB‐FET with Optimized MPB Stack

We validated the performance improvement through capacitance enhancement in a planar MPB‐FET fabricated on a simplified platform before implementing the optimized mixed‐HZO thin film in the NS GAA architecture. Silicon‐on‐insulator (SOI) substrates with a body‐floating configuration similar to NS GAAFETs were utilized. The devices were fabricated with dimensions [channel width (*W* = 10 µm) and gate length (*L*_G_ = 0.5 µm)] to extract all the *P*–*V*, *I*–*V*, and *C*–*V* characteristics from the same device for the rigorous analysis of the physical origin of performance enhancement (Figures S3a,b, Supporting Information). Figure 1g shows the hysteretic drain/gate current (*I*_D_/*I*_G_)–gate voltage (*V*_G_) characteristics at a drain voltage (*V*_D_) of 50 mV for devices with pure HfO and mixed HZO 1 stacks, where the gate current was found to be sufficiently low, as confirmed by the MFIS experiments. The enlarged graph (Inset of Figure 1g) shows a nearly hysteresis‐free nature within the typical logic operation range due to the stable MPB material design. It was confirmed that our optimized MFIS gate stack ultimately exhibits hysteresis characteristics that are independent of sweep time. Furthermore, even under very slow DC sweep conditions (≈0.6 s V^−1^), the hysteresis remains minimal (less than 10 mV) within a ±1 V sweep range (Figure S4, Supporting Information). In contrast, the AFE (ZrO_2_) device exhibited counterclockwise hysteresis in the *V*_TH_ region (Figure 1h), where the forward‐swept *SS* was lower, while the reverse‐swept *SS* was comparable to that of the MPB device (Figure S5, Supporting Information). This is attributed to the abrupt polarization switching (field‐induced nonpolar‐to‐polar switching) of the antiferroelectric material in the forward sweep and polar‐to‐nonpolar reversal switching ≈0 V, as shown in the inset of Figure 1h. These findings highlight the importance of a refined material design in Zr‐doped HfO_2_ system.

**Figure 2:**
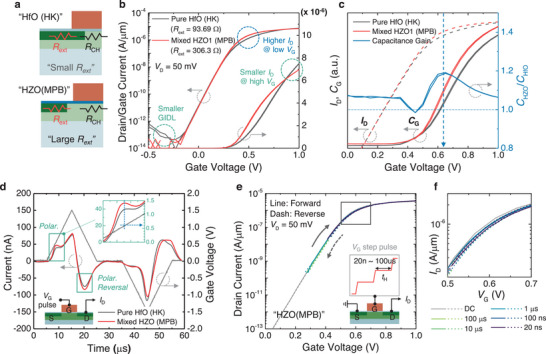
Electrical characteristics of SOI planar MPB‐FETs and capacitance boosting effect by stabilized MPB. a) Illustration of two devices with differently designed *R*_ext_ (see Note S2, Supporting Information for details). b) *I*_D_–*V*_G_ curves of planar FETs (HfO (HK) and HZO (MPB)) with distinct *R*_SD_. Here, smaller GIDL current and *I*_D_ (@ high *V*_G_) indicate the variance of the *R*_SD_ resulting from different S/D overlaps. c) *C*_G_–*V*_G_ and *I*_D_–*V*_G_ (left‐y), and *C*_G_ ratio of HZO over HfO_2_ device (right‐y) measured from the planar FETs. d) Displacement current and *V*_G_ measured from the planar FETs and experiment setup for a triangular *V*_G_ pulse measurement on the planar FETs (Figure S5, Supporting Information). (inset) Enlarged graph shows enhanced *C*_G_ in the vicinity of the *V*_TH_ (≈0.6 V) for the HZO device. e Transient‐measured *I*_D_–*V*_G_s of planar HZO device at various *t*_H_s and experiment setup for a fast *I*_D_–*V*_G_ measurement (the limit of the equipment *t*_H_ = 20 ns). f) Enlarged fast *I*_D_–*V*_G_ graph, compared to DC transfer characteristic.

Note that the mixed HZO device exhibited an increased *I*_on_ in the vicinity of *V*_TH_, along with a slightly reduced *SS* compared to the pure HfO device, which is likely due to the capacitance boosting effect occurring immediately after *V*_TH_. Nevertheless, it is reasonable to question whether the difference in *I*_on_ is solely attributable to the variance in the external resistance (*R*_ext_) rather than to the gate capacitance (*C*_G_) boost of the mixed HZO. **Figure** **2**a shows the experimental setup used to answer this question. The source/drain extension resistance was designed to be smaller in the HfO device than in the HZO device through intentional junction control (Figure S3c,d and Note S2, Supporting Information). As shown in Figure 2b, the HfO device (with a smaller *R*_ext_) exhibited a higher *I*_D_ in the high *V*_G_ region, where the current was predominantly affected by *R*_ext_. Additionally, the HfO device showed a higher gate‐induced drain leakage (GIDL) resulting from the enlarged overlap region. The extracted on‐resistance (Figure S3e, Supporting Information), where the *R*_ext_ of the HfO device (93.69 kΩ) was found to be lower than that of the HZO device (306.3 Ω), is precisely consistent with the current data. Nevertheless, the current increase in the HZO device (with a larger *R*_ext_) was particularly pronounced within a small overdrive voltage (*V*_OD_) range, clearly revealing that the current enhancement of the HZO device was intrinsically due to *C*_G_ amplification. The mixed HZO enhanced the current by 25–50% compared to the pure HfO stack at various *V*_D_s and *V*_OD_s (**Table** **1**). Here, a *V*_OD_ of 0.35 V approximately corresponds to the operation *V*_G_ considering the *V*_TH_ and *V*_DD_ of 1.0 nm eq node high density (HD) target.^[^ ^40^ ^]^ Notably, *I*_D_ enhancement was particularly prominent at low *V*_OD_, indicating the superiority of the HZO device for low‐power applications. This phenomenon can be attributed to 1) the increased impact of *R*_ext_ in the high *V*_OD_ range and/or 2) capacitance boosting being particularly emphasized in the lower *V*_OD_ region.

**Table 1:** On‐current enhancement of planar HZO (MPB) device over pure HfO_2_ (HK) device.

Overdrive voltage [V]	On‐current enhancement over pure HfO_2_ device	
	*V*_D_ = 50 mV	*V*_D_ = 0.5 V
0.25	46.59%	51.34%
0.35	33.35%	43.10%
0.45	24.84%	35.67%

The following analyses clarified *I*_D_ amplification at low *V*_OD_. Figure 2c illustrates the small‐signal *C*_G_s directly measured from the two FETs (left y) and their ratios (right y) along with the corresponding *I*_D_s (left y), showing a capacitance boost near the *V*_TH_ region. This phenomenon can be explained by the fact that at the onset of channel inversion, the potential begins to be predominantly applied to the HZO layer, inducing additional inversion charges (i.e., *I*_D_ enhancement) via polarization switching.^[^ ^29^ ^]^ In addition to the stabilized MPB in the mixed‐phase HZO, capacitance boosting can also be understood in terms of the stray electric field (*E*_S_) between the domains (Figure S6, Supporting Information). Under a bias of 0 V at the HZO–SiO_2_ interface, *E*_S_ lines were observed between the domains. When a low voltage of 0–1 V was applied, the *E*_S_ lines transformed into out‐of‐plane electric field (*E*_OP_) components. This *E*_OP_ plays a crucial role in compensating for the additional charge at the HZO–SiO_2_ interface, resulting in an elevated permittivity response in mixed‐phase HZO.^[^ ^28^, ^41^ ^]^ This effect is particularly pronounced in scaled HZO, where a dense domain pattern is formed in an ultrathin ferroelectric layer. This leads to a more extensive transformation of the *E*_S_ into an *E*_OP_.

Further support was provided by triangular *V*_G_ pulse measurements (quasi‐static *C*–*V* measurements) from the n‐type FETs, as illustrated in Figure 2d (see Figure S7a, Supporting Information for detailed measurement setup). Considering the operating voltage of the logic device, a triangular *V*_G_ pulse of up to 1.5 V at a speed of 1.5 V/10 µs was applied, and the transient *I*_D_ was measured. Here, the measured transient *I*_D_ directly reflects *C*_G_ (Refer to Figure S7b, Supporting Information). Both polarization (rising) and polarization reversal (falling) switching peaks were observed in the vicinity of the *V*_TH_ region (at almost the same *V*_G_ of ≈0.6 V) in the HZO device. In contrast, a conventional inversion *C*–*V* curve without polarization switching appeared in the HfO device. All previous studies have demonstrated enhanced small‐signal capacitance as an indicator of stabilized NC.^[^ ^28^, ^29^ ^]^ Higher permittivity can be obtained without a stabilized NC from the charge compensation caused by the out‐of‐plane electric field between the domains in the mixed‐phase HZO. Thus, the observation of polarization/polarization reversal switching currents at almost the same *V*_G_ in quasi‐static *C*–*V* measurements is a direct and robust proof of hysteresis‐free stabilized NC.

It is imperative that the validation of MPB‐FETs intended for high‐speed logic applications extend beyond DC–*I*_D_ analyses. Most previous studies, except those performed by Kwon et al., have not delved into speed analyses.^[^ ^12^ ^]^ To completely verify the stabilized NC instead of the transient NC, which is observed by the delay in the transient polarization switching, the transient current characteristics were comprehensively examined through fast *I*_D_–*V*_G_ sweeping, as shown in the inset of Figure 2e.^[^ ^42^ ^]^ Here, the stepwise *V*_G_ pulses (0–1 V) were applied, and the resulting *I*_D_ was averaged within the hold time (*t*_H_), which was varied from 20 ns (minimum applicable period) to 100 µs. As indicated in Figure 2e and its enlarged plot (Figure 2f), the transfer curves are comparable to those of the DC‐measured current despite the slight variation in *V*_TH_ with speed. This confirms that capacitance boosting by the HZO layer remains static regardless of the operation speed (at least up to several tens of nanoseconds).

### 2.4 Channel Stacked NS MPB‐FET

**Figure 3:**
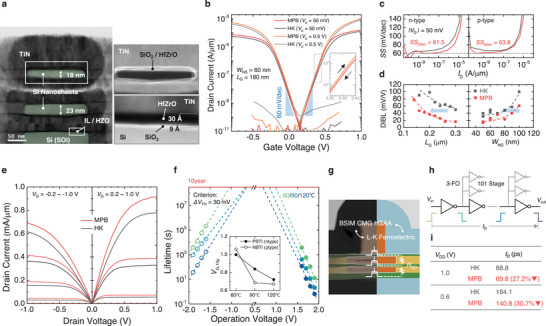
Structure of GAA‐NS MPB‐FETs and the electrical characteristics. a) Cross‐sectional TEM images of fabricated 2‐stacked NS MPB‐FET and enlarged TEM images of the 2nd (top) Si NS channel and gate stack configuration on bottom SOI. b) *I*_D_–*V*_G_ curves of fabricated 2‐stacked NS GAA FETs (HK & MPB device) and (inset) enlarged dual‐swept graphs of n‐type MPB device. c) *SS*‐*I*_D_ plots of (left) n‐ and (right) p‐type devices. d) *DIBL* of n‐type NS GAAFETs depending on (left) the gate length and (right) the nanosheet width. e) Output characteristics of (left) p‐ and (right) n‐type NS GAAFETs. f) N/PBTI lifetime plots (Δ*V*_TH_ = 30 mV criterion) and (inset) extrapolated operating gate voltages for ten years (*V*_G,10y_). g) Schematic image that illustrates the compact model of stacked NS GAA MPB‐FET aligned with the cross‐sectional (channel‐direction) TEM image. h) Schematic image of 101‐stage 3‐FO inverter chain simulation and i) summarized results: extracted *t*_D_s of HK and MPB GAA devices for *V*_DD_ = 1 and 0.6 V.

To examine the feasibility of the mixed‐phase MPB HZO material in stacked NS technology, we fabricated stacked NS GAA MPB‐FETs via a gate‐first process. The detailed sequences are provided in Figure S8 and Note S3 (Supporting Information). **Figure** **3**a displays the cross‐sectional TEM images of the fabricated 2‐stacked NS GAA MPB‐FET, highlighting the vertically stacked Si NS channels released above the SOI channel. The magnified TEM image (Figure 3a) shows the ALD gate stack comprising SiO_2_ (9 Å) and HZO (30 Å). According to 2D material mapping data obtained through EDS analysis, the ALD gate stack entirely enveloped the released NS channels (Figure S8g, Supporting Information). Furthermore, 1D (line) atomic percentage data from EDS analysis (Figure S8h, Supporting Information) confirmed the successful removal of SiGe layer via adequate selective etching, with Hf and Zr contents measured at ≈25% and 75%, respectively, within the HZO stack.^[^ ^43^ ^]^

Figure 3b illustrates the transfer characteristics of the co‐integrated n‐ and p‐type NS GAA MPB‐FETs [nanosheet width (*W*_NS_) = 60 and *L*_G_ = 180 nm] at *V*_D_ = 50 mV/0.5 V, compared to those of the reference (HfO_2_) NS GAAFET. The MPB devices exhibited nearly hysteresis‐free behavior, attributed to the MPB stabilization in the mixed‐phase HZO film, as depicted in the inset of Figure 3b. Due to the absence of sufficient body doping and/or WF engineering, a mismatch in *V*_TH_s of ≈0.18 V was observed between the n‐ and p‐type devices. Yet, similar to the results of the SOI planar FET discussed in the previous section, current enhancements were most pronounced near *V*_TH_ in the NS MPB devices, demonstrating that the field‐dependent current‐boosting effect of the optimized MPB stack is well‐suited for the NS GAA structure. Figure 3c compares the *SS* of p‐/n‐type MPB and HK devices with respect to *I*_D_, which confirms the *SS* improvement in MPB devices due to the slightly enhanced permittivity and capacitance boosting in the subthreshold region (*C*_HZO_/*C*_HfO_ > 1 in Figure 2c). The well‐designed MPB material, combined with the NS GAA structure achieved an average *SS* over three decades (*SS*_3‐dec_) as low as 61.5 (n‐type) and 63.8 (p‐type) mV dec^−1^, approaching the thermionic limit.

The *I*_on_ gains measured at various *V*_OD_s are summarized in **Table** **2**, demonstrating 24.2% enhancement at *V*_OD_ = 0.35 V compared to HK devices. These improvements, combined with the better *SS*, allow for a *V*_G_ reduction to acquire the same drivability as the HK device. This is particularly significant as it demonstrates the potential for performance enhancement without increasing the *I*_off_, while also simplifying the complex work function metal (WFM) process, which poses considerable challenges in the NS fabrication.^[^ ^44^, ^45^ ^]^ Additionally, the *V*_G_ scalability of the MPB device, compared to the reference HK device was confirmed to be 82.5 mV at *V*_OD_ = 0.35 V, which is a notable improvement given the adversity associated with WF modulation.

**Table 2:** On‐current enhancement and gate voltage scalability of NS GAA MPB‐FET over pure HfO_2_ device.

Overdrive voltage [V]	@ *V*_D_ = 50 mV	
	*I*_on_ enhancement	*V_G_* scalability
0.25	29.3%	65.1 mV
0.35	24.2%	82.5 mV
0.45	23.3%	109 mV

Figure 3d presents the drain‐induced barrier lowering (DIBL) characteristics of the MPB and HK devices in relation to the *L*_G_s and *W*_NS_s, where severe SCE degradation is observed with decreasing *L*_G_ and increasing *W*_NS_.^[^ ^6^ ^]^ However, EOT scaling, driven by the capacitance‐boosting effect of the MPB HZO, successfully mitigated SCE. The *I*_D_–*V*_D_ characteristics of the p‐ and n‐type NS GAAFETs, shown in Figure 3e, reveal current enhancement across the entire *V*_D_ range in the MPB devices. Additional data on the current behavior of the NS GAA MPB‐FETs depending on *W*_NS_ and *L*_G_ can be found in Figure S9 (Supporting Information).

The bias temperature instability (BTI) characteristics, including both negative BTI (NBTI) and positive BTI (PBTI), were investigated for the p‐ and n‐type NS GAA MPB devices (Figure 3f) to ensure the reliability of the designed thin film. Here, the lifetime under *V*_G_ stress at 60, 90, and 120 °C is defined based on the failure criterion of ∆*V*_TH_ = 30 mV. The detailed procedure for determining BTI lifetime is provided in Figure S10 (Supporting Information). The operating voltages of MPB devices that satisfy the 10‐year lifetime (*V*_G,10y_) are plotted in the inset of Figure 3f, demonstrating that both n‐ and p‐type devices ensure acceptable BTI characteristics for low *V*_DD_ applications, specifically 0.6 V, in line with the IEEE International Roadmap for Devices and Systems (IRDS) 1.0 nm eq node HD specifications).^[^ ^40^ ^]^

To predict the AC characteristics of the fabricated devices, an inverter chain circuit simulation was performed using a carefully fitted BSIM–CMG compact model with an embedded Landau‐Khalatnikov (L–K) ferroelectric model. As shown in Figure 3g, to mimic a vertically stacked GAA FET, horizontal GAA (hGAA) models were connected in parallel with *R*_SD_s between them. The L–K ferroelectric model with fitted ferroelectric parameters was utilized to calculate the gate charge to be delivered to the BSIM–CMG model (Figure S11a and Note S4, Supporting Information). The *I*_D_–*V*_G_ curves at *V*_D_ = 50 mV and 0.5 V, along with the *C*_G_–*V*_G_ curves showed good agreement with our model, as illustrated in Figure S11b–d (Supporting Information). Next, 101‐stage CMOS inverter chains with three‐fanout (FO) were simulated, as depicted in Figure 3h. The calculated propagation delays (*t*_d_) of the MPB and HK inverters are summarized in Figure 3i, where the MPB CMOS inverter exhibits 27.2% and 30.7% reductions in *t*_d_ compared to the HK inverter at *V*_DD_ = 1 and 0.6 V, respectively. This emphasizes the low‐*V*_DD_ benefit of NS MPB‐FETs for AC operation.

In Figure S12a–d (Supporting Information), the measured *I*_D_–*V*_G_ curves for the NS GAA MPB‐FETs with various *L*_G_ values are benchmarked to the vertically stacked nanowire MOSFETs of the IMEC (see Note S5, Supporting Information).^[^ ^46^ ^]^ The *I*_on_–*I*_off_ was determined by shifting the *I*_D_–*V*_G_ curve to different *I*_off_ values and evaluating *I*_on_ at *V*_G_ = *V*_D_ = *V*_DD_. The n‐type NS GAA MPB‐FETs showed comparable *I*_on_‐*I*_off_ properties at *V*_D_ = 0.9 V to those of the IMEC counterparts (Figure S12a, Supporting Information) despite having a significantly longer *L*_G_. Additionally, Figure S12b (Supporting Information) shows that the n‐type NS GAA MPB‐FETs exhibited superior *I*_on_–*I*_off_ with increasing *V*_D_ because they had a relatively large series resistance on the source and drain sides, and the effect of *R*_SD_ on *I*_on_ was suppressed at a larger *V*_D_. However, for the p‐type NS GAA MPB‐FETs, the *I*_on_–*I*_off_ trend is compatible from *V*_D_ = 1.0 V (Figure S12d, Supporting Information) because the p‐type MPB‐FETs have the larger *R*_SD_. Figure S12e–f (Supporting Information) shows the extrinsic and intrinsic transconductance (*g*_m_) for n‐ and p‐type NS GAA MPB‐FETs with various *L*_G_s measured at various *V*_D_s, respectively. The measured intrinsic transconductance (*g*_m,i_) is benchmarked in Figure S12g,h (Supporting Information) against previous reports of n‐ and p‐type MOSFETs with HfO_2_‐based conventional HK gate stacks.^[^ ^46^, ^47^ ^]^ With the mixed‐phase HZO gate stack, *g*_m,i_ exhibited similar *L*_G_ scaling trends when normalized by the effective width (*W*_eff_) and was higher than the overall *g*_m.i_‐*L*_G_ trend with conventional HK gate stacks when normalized by the footprint (i.e., *W*_NS_). Thus, the increase in transconductance is attributed to the fact that the enhanced capacitance of the mixed‐phase HZO gate oxide did not result in any deterioration of the electron/hole transport. The direct comparison of effective mobility (*µ*_eff_) between the HK and MPB devices on our stacked NS GAA platform is summarized in **Table** **3**.

**Table 3:** Comparison of *µ*_eff_ between the HK and MPB devices on our stacked NS GAA platform. Refer to Note S5 (Supporting Information) for detailed extraction method. Due to the improved EOT of the MPB thin film, the effective surface potential (*ψ*_s_) at the same gate voltage is higher, leading to a slightly larger mobility. The comparison results verify that the MPB does not degrade mobility despite the effective reduction in EOT.

Device	Effective electron mobility (n‐type)	Effective hole mobility (p‐type)	*N*_inv_ @ *V*_G_ = 1 V
HK (pure HfO)	182.48 cm^2^ Vs^−1^	218.17 cm^2^ Vs^−1^	3.18 × 10^12^ cm^−3^
MPB (mixed HZO)	158.13 cm^2^ Vs^−1^	198.27 cm^2^ Vs^−1^	3.36 × 10^12^ cm^−3^

Finally, Table S2 (Supporting Information) provides a comprehensive comparison of the performance metrics for various reported ferroelectric‐FETs configurations, including NCFETs (see Note S7, Supporting Information for the performance extraction method). Considering the state‐of‐the‐art GAA structure, EOT of the gate insulator, hysteresis‐free operation, comparable *I*_ON_ to industry logic devices, and *SS* approaching the thermionic limit, the NS GAA MPB‐FETs align well with the technology roadmap for advanced low‐power and high‐performance logic applications.

## 3 Conclusion

We confirmed the capacitance‐boosting effect of FE–AFE mixed‐phase HZO and demonstrated two‐stacked NS GAA MPB‐FETs. The MPB HZO stack was designed based on capacitance and material analyses conducted using MFM and MFIS capacitors. Through DC *I*_D_ measurements of the fabricated planar SOI devices, it was confirmed that *I*_D_ was enhanced, particularly near or above *V*_TH_ without hysteresis in the mixed HZO stack device, which was attributed to the capacitance‐boosting effect verified by small‐signal *C*_G_ measurements. The polarization/polarization reversal switching current observed in the quasi‐static *C*_G_ analyses proved that the hysteresis‐free capacitance boosting of the mixed HZO stack originated from the stabilized NC, along with the charge compensation caused by the out‐of‐plane electric field between the domains. Additionally, the transient *I*_D_–*V*_G_ results confirmed that *I*_on_ enhancement by capacitance boosting was consistent, regardless of the speed.

The carefully designed MPB material was, as a pioneering effort, integrated into an advanced logic technology, the two‐stacked NS GAAFETs, demonstrating performance enhancement in low *V*_OD_ and SCE immunity and feasible BTI properties. Moreover, the predicted inverter performance highlights the superiority of NS MPB devices over HK devices especially for low‐voltage operation. The transconductance benchmark of our NS GAA MPB‐FET, which is similar to that of industrial planar devices, revealed that the EOT scaling of the mixed HZO did not lead to any mobility degradation. We identified the physical origin of EOT scaling in a mixed HZO MPB stack and verified the potential of using this material in advanced low‐power and high‐performance NS technology to overcome the power consumption limitation of high‐k technologies. Our work demonstrated that the HfO_2_‐based MPB material, with its excellent process compatibility and low thermal budget (500 °C), offers a promising solution for seamless integration into logic mass production without incurring significant development costs.

## 4 Experimental Section

### MFM Capacitor Fabrication

The MFM capacitors were fabricated as follows: The substrate was cleaned using SC‐1 (NH_4_OH:H_2_O_2_:H_2_O = 1:1:5) and buffered oxide etchant (BOE, NH_4_F:HF = 6:1) wet etching. A 50 nm TiN layer was deposited via DC sputtering as the bottom electrode. Then, HZO with a thickness of 6 nm was deposited by atomic layer deposition (ALD) at 330 °C using TEMA‐Hf, TEMA‐Zr, and O_3_ as the precursors and oxygen source, respectively. The deposition cycle included 1) repeated single cycle of HfO_2_ and ZrO_2_ for the FE, 2) four cycles of ZrO_2_ followed by two cycles of HfO_2_ for the MPB, and 3) only ZrO_2_ cycles for the AFE. A second TiN bilayer was deposited sequentially using ALD and DC sputtering to form the top electrode, followed by patterning. Rapid thermal annealing (RTA) was conducted for orthorhombic phase formation under N_2_ ambient at 500 °C for 30 s.

### MFIS Capacitor Fabrication

An MFIS capacitor with a self‐aligned doping region serving as an inversion electron reservoir (Figure S2a, Supporting Information) was fabricated using the following steps (Figure S2b, Supporting Information). First, interfacial SiO_2_ (15 cycles), followed by several types of HZO and HfO stacks, was deposited on a p‐type wafer through ALD, each with an identical thickness of 30 Å. As a high‐κ control, 40 cycles of pure HfO were deposited, while two types of Zr‐rich HZO stacks (Mixed HZO 1 and Mixed HZO 2) were deposited to achieve a ferroelectric‐antiferroelectric mixed‐phase. The HZO stack 1 is identical to those verified by the MFM capacitor experiments (Figure S1b, Supporting Information) but with reduced thickness for practical logic applications. For Mixed HZO 1, ZrO_2_ and HfO_2_ were deposited in a solid solution, alternating between four cycles of ZrO_2_ and two cycles of HfO_2_. Mixed HZO 2 was deposited in a nanolaminate form in the order of HfO 5 cycles, ZrO_2_ 24 cycles, and HfO_2_ 5 cycles. Subsequently, TiN deposition was performed using a combination of ALD and sputtering, followed by gate patterning through photolithography and inductively coupled plasma reactive ion etching (ICP‐RIE) dry etching using Cl_2_ gas. To evaluate the inversion characteristic of the MFIS capacitor, a self‐aligned n+ doping was performed using As^+^ at 20 keV and a dose of 2 × 10^15^ cm^−2^ with a medium current ion implanter. HZO crystallization and dopant activation were achieved through a PMA process at 500 °C for 30 s using rapid thermal annealing (RTA). Lastly, to minimize the interfacial trap density (*D*_it_), a high‐pressure annealing (HPA) process was conducted in an H_2_ atmosphere (18 Bar, 400 °C, 1 h).

Figure S2c (Supporting Information) shows the *C*–*V* characteristics of a MOS (TiN‐HfO_2_‐SiO_2_‐p‐ Si) capacitor with and without HPA. The reduction in the hump in the *C*–*V* curve after HPA indicated a significant decrease in *D*_it_, which can be attributed to the elimination of dangling bonds at the Si‐SiO_2_ interface. *C‐*‐*V* measurements were performed using an LCR meter (HP Agilent 4284A) at a frequency of 1 kHz. The resulting *C*–*V* curve of the fabricated MFIS capacitor is shown in Figure S2d (Supporting Information), where the inversion capacitance is clearly observed, facilitated by the self‐aligned n^+^ electron reservoir.

### Electrical Measurements

The ferroelectricity of the capacitors was investigated using a parameter analyzer (Keithley 4200‐SCS) and a current‐voltage module (4225‐PMU). The *P*–*V* curves were measured using a positive‐up‐negative‐down (PUND) method in conjunction with a time‐transient measurement using a triangular pulse with a frequency of 2.5 kHz. In the PUND measurements, ferroelectric switching currents were obtained and calculated from the difference between positive‐up and negative‐down because the currents of positive/negative pulses include both switching (ferroelectric switching) and nonswitching (displacement and leakage) components, and the currents of up/down pulses have only nonswitching components.

The direct current (DC) *I*_D_–*V*_GS_ measurements were performed using a semiconductor parameter analyzer (Keysight B1500A). A B1500A equipped with a waveform generator/fast measurement unit (WGFMU) module was used for the pulsed *I*–*V* measurements. For quasi‐static *C*–*V* measurements, the WGFMU module was used to supply a triangular voltage to the gate, and the transient drain current was measured using a current oscilloscope. Small signal *C*–*V* measurements were performed using an LCR meter (HP Agilent 4284A) at a frequency of 1 kHz.

## Conflict of Interest

The authors declare no conflict of interest.

## Supporting information



Supporting Information

## Data Availability

The data that support the findings of this study are available from the corresponding author upon reasonable request.
