# Supplementary material for: Stacked Nanosheet Gate‐All‐Around Morphotropic Phase Boundary Field‐Effect Transistors
Source: Adv Sci (Weinh). 2025 Mar 17;12(18):2413090. doi: 10.1002/advs.202413090 (PMC12079460; doi:10.1002/advs.202413090)
Supplement: Supplementary file 1 — Supporting Information [file ADVS-12-2413090-s001.pdf]

ADVANCED SCIENCE
Open Access

Supporting Information

for *Adv. Sci.*, DOI 10.1002/advs.202413090

Stacked Nanosheet Gate-All-Around Morphotropic Phase Boundary Field-Effect Transistors

Sihyun Kim, Hyun-Min Kim, Ki-Ryun Kwon and Daewoong Kwon**

Supporting Information

Stacked Nanosheet Gate-All-Around
Morphotropic Phase Boundary Field-Effect Transistors

Sihyun Kim[2†], Hyun-Min Kim[1†], Ki-Ryun Kwon[1] and Daewoong Kwon[1]**

[1] Department of Electrical Engineering, Hanyang University, Seoul 04763, Republic of Korea
[2] Department of Electronic Engineering, Sogang University, Seoul 04107, Republic of Korea

[†] These authors contributed equally: Sihyun Kim, Hyun-Min Kim
** Corresponding author (e-mail: skim@sogang.ac.kr, dw79kwon@hanyang.ac.kr)*

Contents

Supporting Information Figure S1–S12

Supporting Information Table S2

Supporting Information Note S1–S7

Supporting Information References

Supporting Information Figures

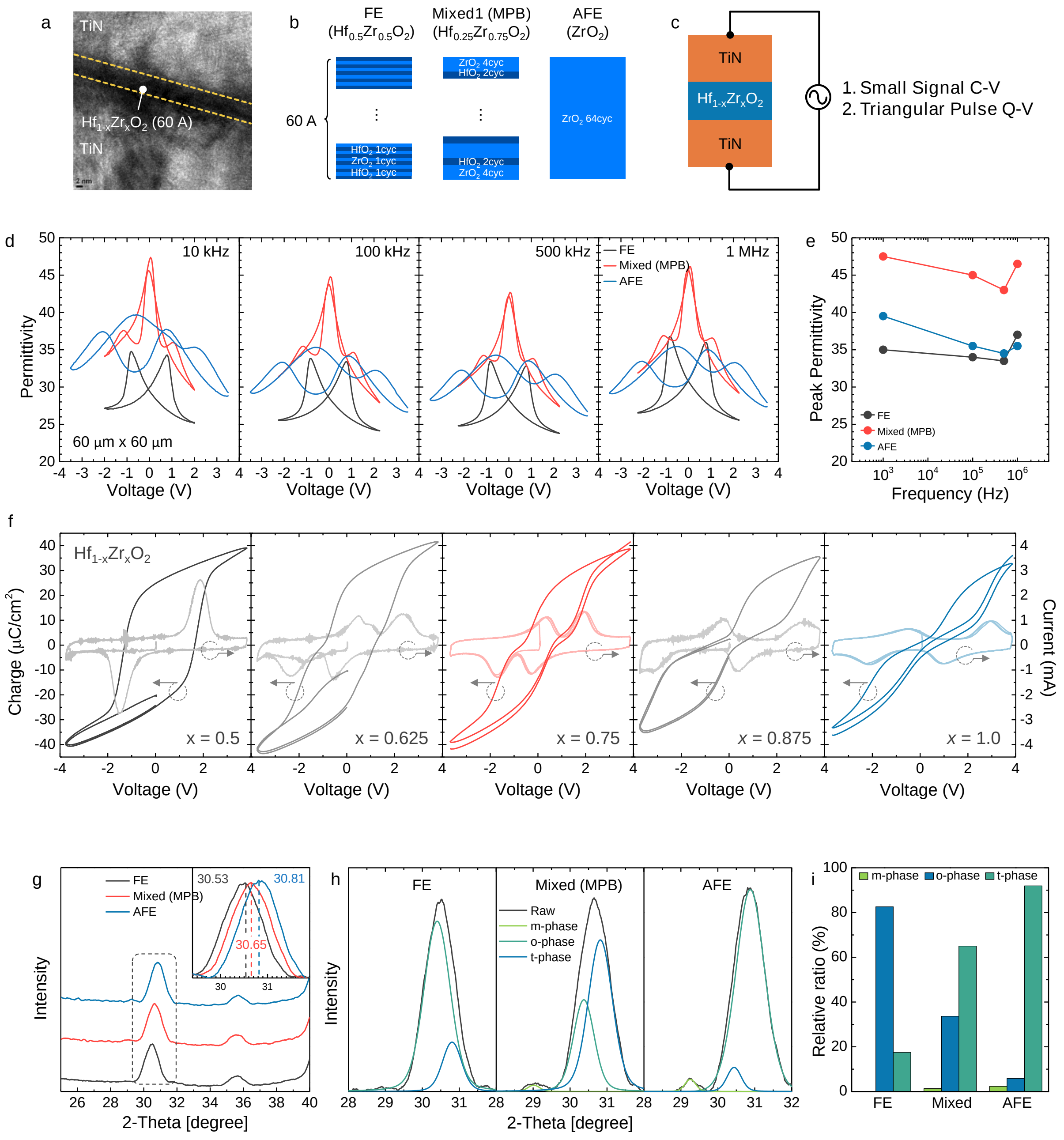

Supporting Information Figure S1. Ferroelectric material optimization in MFM capacitors. a TEM image of fabricated MFM capacitor (mixed HZO). **b** Material design for FE ($Hf_{0.5}Zr_{0.5}O_2$), Mixed ($Hf_{0.25}Zr_{0.75}O_2$), and AFE (ZrO_2) ALD films (equally 60 Å). **c** MFM capacitor measurement setups: the small signal *C-V* and triangular pulse *Q-V*. **d** Small signal *C-V* butterfly curves of MFM capacitors with various *x* measured with different AC frequencies (10 kHz, 100 kHz, 500 kHz and 1 MHz), exhibiting signatures of FE (x=0.5)/Mixed

(x=0.75)/AFE (x=1) phases. **e** Peak permittivity versus AC frequency plots for the three compositions, demonstrating that the MPB film consistently exhibits the highest permittivity (~45.5) across all frequencies. **f** Triangular pulse measurements [*Q*-*V* (left-y) and *I*-*V* (right-y)] of fabricated $Hf_{1-x}Zr_xO_2$ MFM capacitors (x=0.5, 0.625, 0.75, 0.875, 1.0), demonstrating FE-mixed-AFE hysteretic curves. **g** GIXRD patterns and **h** deconvoluted GIXRD peaks of FE/Mixed/AFE capacitors (**Supporting Information Note S1**). **i** Relative phase ratio of the capacitors with three different compositions.

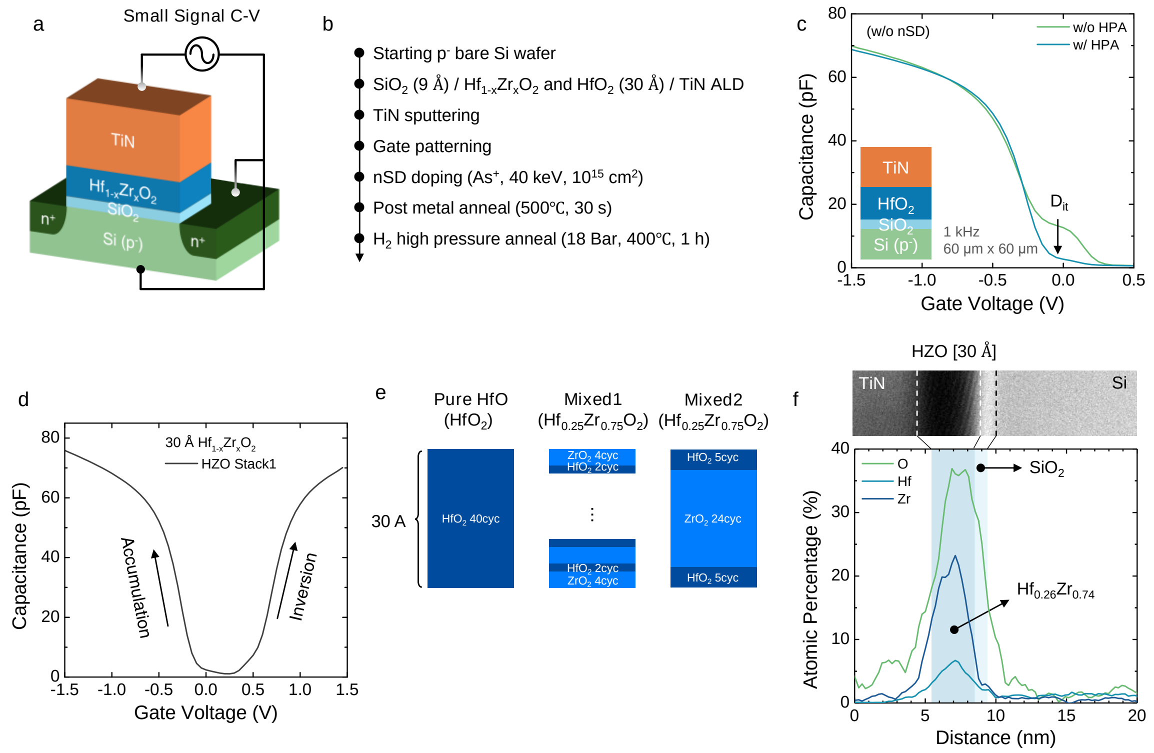

Supporting Information Figure S2. Fabrication of MFIS capacitors. a Schematic image of MFIS capacitor with self-aligned n-doping region for electron reservoir and its *C-V* measurement configuration. **b** Summarized process flow of MFIS capacitor fabrication. Note that 1) nSD doping to inspect the inversion capacitance and 2) H_2 high-pressure annealing (HPA, 18 Bar, 400°C for 1 hour) for D_{it} reduction were included. **c** *C-V* characteristics of MOS (TiN-HfO_2-SiO_2-p-Si) capacitor with and without HPA process. The lowering of the hump in the *C-V* curve in the MOS capacitor with HPA confirms that the D_{it} was reduced due to the elimination of the Si-SiO_2 interfacial dangling bond. **d** *C-V* curve of MFIS capacitor with electron reservoir (nSD), exhibiting both accumulation and inversion region. **e** Material design for ALD stacks for Pure HfO (HfO_2), Mixed 1, and 2 ($Hf_{0.25}Zr_{0.75}O_2$) capacitors. Mixed 1 was composed of repeatedly deposited ZrO_2 (4 cycles) and HfO_2 (2 cycles): solid solution, while Mixed 2 was made up of 24 cycles of ZrO_2 sandwiched by five cycles of HfO_2: nano-laminated configuration. **f** 1D line EDS analysis (Atomic %) of fabricated capacitor.

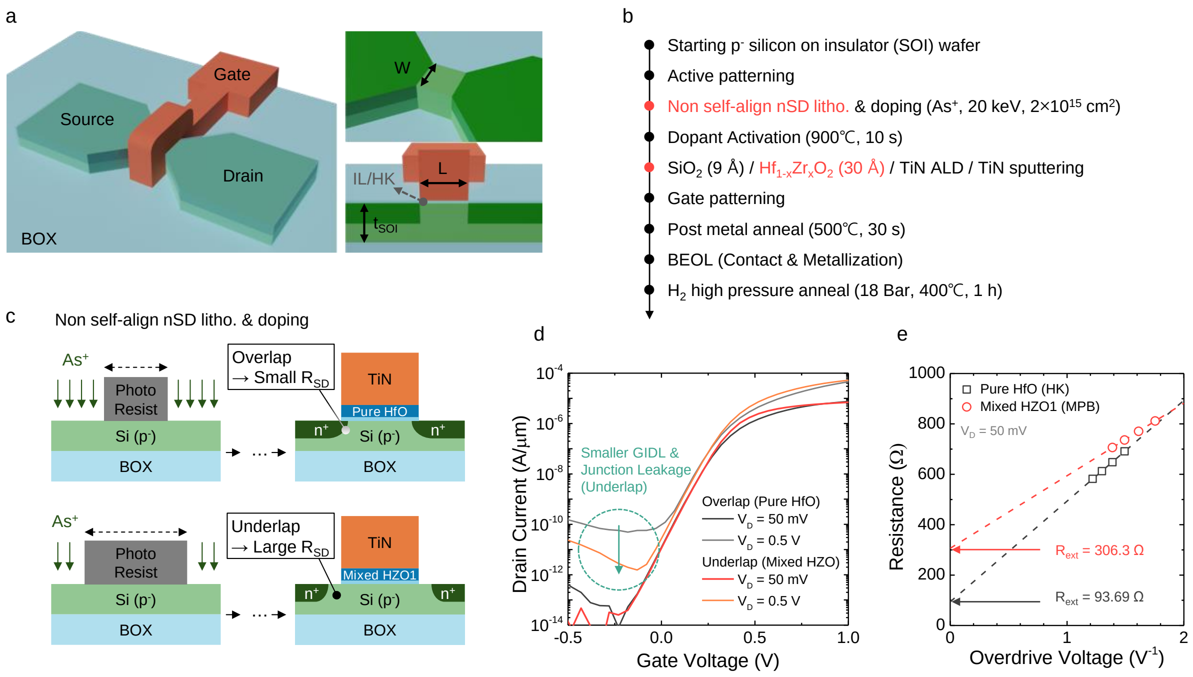

Supporting Information Figure S3. Fabrication of SOI planar FETs. a Bird's-eye view of SOI planar FETs. **b** Summarized procedure for fabricating the SOI planar FETs: Mixed HZO1 and pure HfO2 devices. Detailed information is described in the Supporting Information Note S2. The non-self-algin S/D doping proceeded for two regions: 1) to fully activate the S/D dopants, which cannot be performed by self-aligned S/D process after HZO/HfO_2 deposition, and 2) to control the S/D extension resistance. **c** Fabrication method for R_{ext} adjustment during the NSA S/D doping step (see Supporting Information Note S2 for details) **d** Transfer characteristics comparison between devices with overlapped (small R_{ext}) and underlapped (large R_{ext}) S/D junctions at various V_{DS}. **e** Extracted external resistance (R_{ext}) of two devices with different S/D junctions.

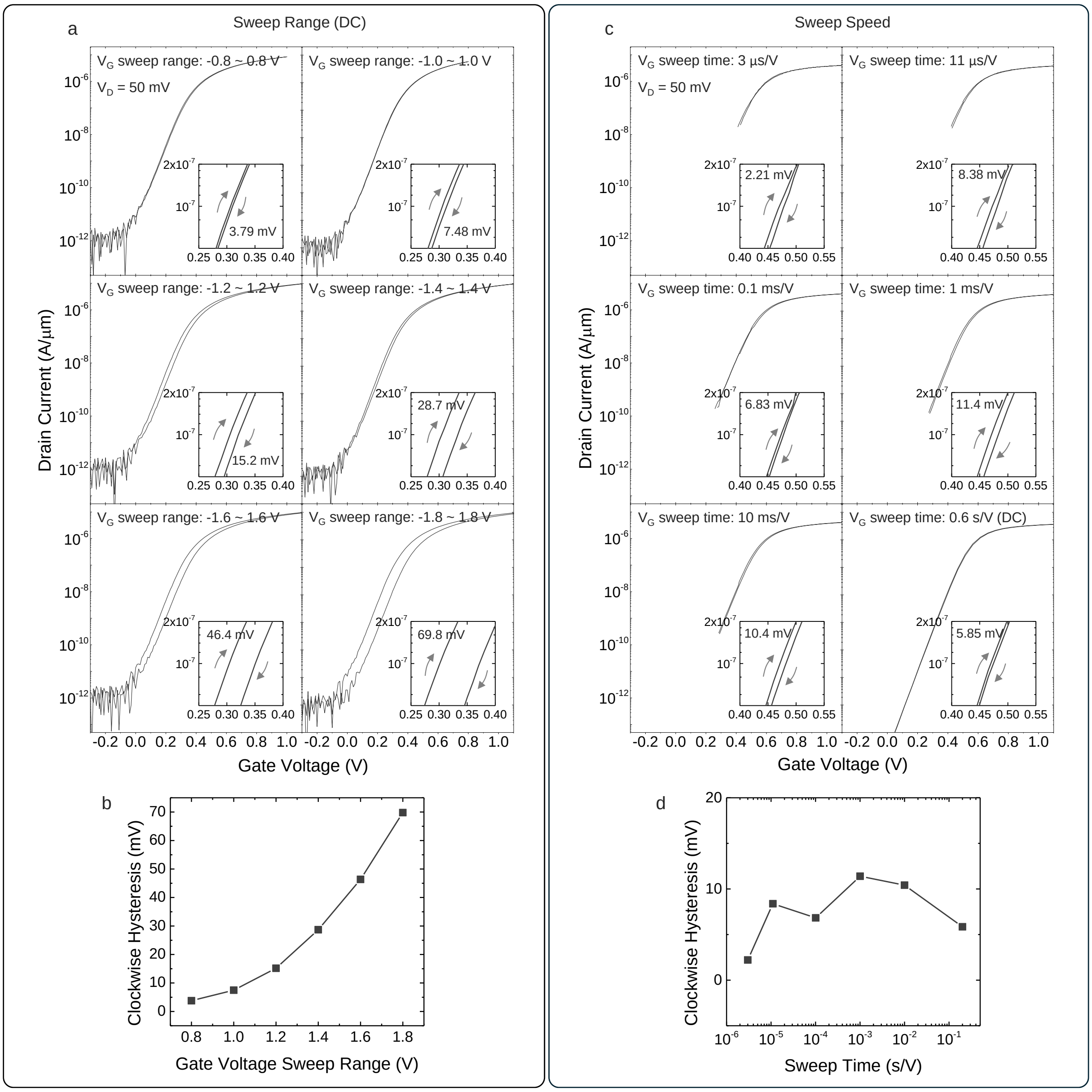

Supporting Information Figure S4. Hysteresis analyses of SOI planar MPB-FETs depending on the V_G sweep range and speed. a Dual swept transfer curves of planar MPB-FETs with sweep ranges of ±0.8 V, ±1.0 V, ±1.2 V, ±1.4 V, ±1.6 V, and ±1.8 V and a sweep time of 0.6 V/s (DC). **b** Summarized clockwise hysteresis regarding the V_G sweep range. **c** Dual swept transfer curves with sweep times of 3 μs/V, 11 μs/V, 0.1 ms/V, 1 ms/V, 10 ms/V, and 0.6 s/V (DC). **d** Summarized clockwise hysteresis regarding the V_G sweep time.

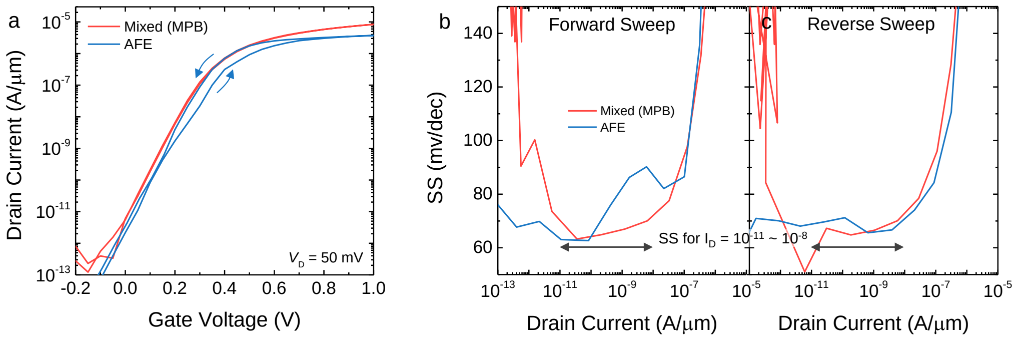

Supporting Information Figure S5. Direct comparison of transfer curves for planar SOI FETs with Mixed HZO1 ($Hf_{0.25}Zr_{0.75}O_2$) and AFE (ZrO_2) FETs. a Transfer characteristics of devices with the two compositions. Here, the V_{TH} was aligned for better comparison. **b-c** Extracted SS-I_D plots from forward- and reverse-swept I_D-V_G measurement, respectively. While the reverse-swept current of the AFE device shows an *SS* value comparable to that of the MPB device, the forward-swept current exhibits significantly lower *SS*. This instability arises from the transient nature of polarization switching in MFIS FETs.[1]

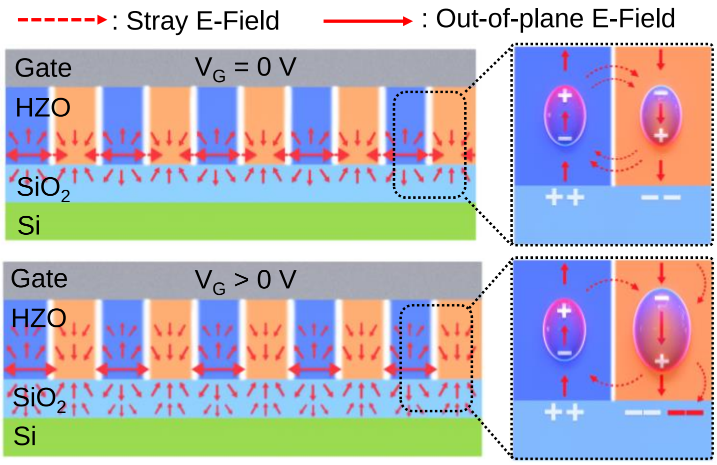

Supporting Information Figure S6. Schematic of electric field (e-field) profile within HZO gate stack in off and on states, respectively. The distribution of stray and out-of-plane e-fields between two adjacent dipoles at $V_G > 0$, describing the origin of higher capacitance in HZO than HfO_2.

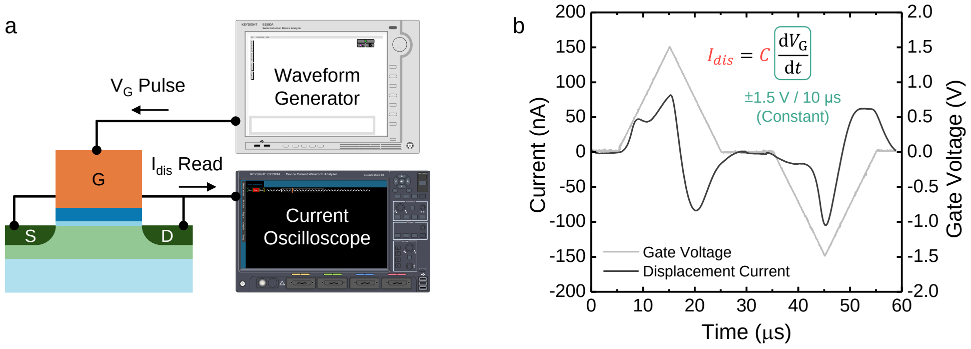

Supporting Information Figure S7. FET-level quasi *C-V* measurement. a FET-level quasi *C-V* measurement setup. Triangular V_G pulse was applied by Keysight B1530A waveform generator/fast measurement unit (WGFMU). At the same time, the corresponding displacement current (I_{dis}) was read by the Keysight CX3324A device current waveform analyzer from the common source/drain. **b** Quasi *C-V* data of planar mixed HZO device (W = 10 μm and L_G = 0.5 μm). Note that the measured I_{dis} directly reflects C_G because I_D is equal to C_G * $\Delta V_G/\Delta t$, where the $\Delta V_G/\Delta t$ is kept constant by the triangular V_G pulse.

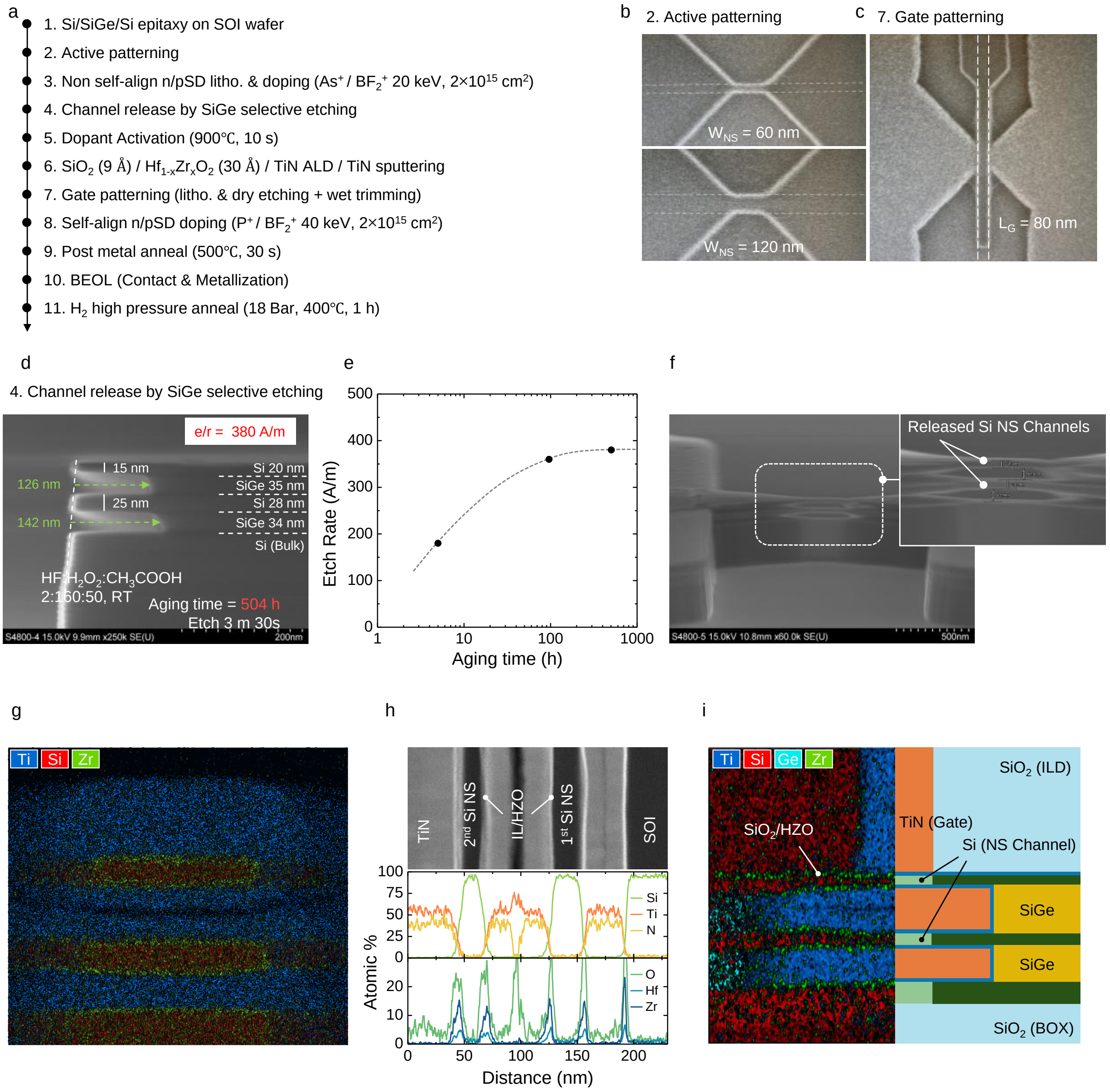

Supporting Information Figure S8. Fabrication and structure of GAA-NS MPB-FETs. a Summarized process flow of 2-stacked NS GAA structured MPB and control HK FET (see Supporting Information Note S3 for details). **b,c** Top-viewed images after the active and gate etching processes observed using in-line critical dimension (CD) SEM equipment. **d** Cross-sectional SEM images test pattern (Si/SiGe multi-epi wafer) after SiGe selective etching using carboxylic acid solution (HF : H_2O_2 : CH_3COOH = 2 : 160 : 50 at room temperature) of which aging time was 504 hours. **e** SiGe etch rate in the solution according to the aging time is stabilized after four days. Details for the SiGe selective etching procedure are illustrated in Supporting Information Note S3. **f** Tilted-viewed SEM image of released NS channels after

active patterning and successive SiGe selective etching using a carboxylic acid solution. **g** Cross-sectional TEM image of fabricated 2-stacked NS MPB-FET (colored by 2D EDS material mapping). **h** Atomic percentage across the GAA stack obtained by 1D (line) EDS analysis. **i** Cross-sectional TEM images of the device across the channel (colored by 2D EDS mapping).

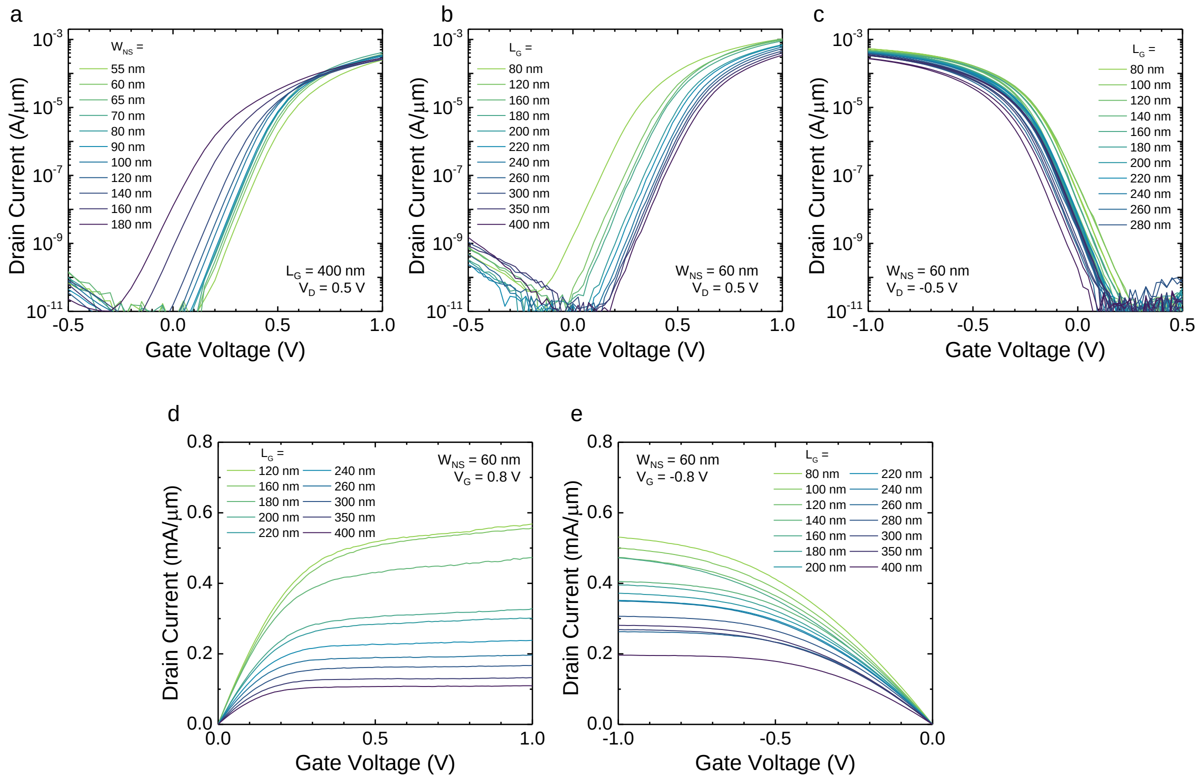

Supporting Information Figure S9. W_{NS} and L_G dependent characteristics of fabricated NS GAA MPB-FETs. a,b I_D-V_G curves of n-type devices with regard to the W_{NS} and L_G, respectively. **c** I_D-V_G curves of p-type devices as a function of L_G. The *SS* degraded as W_{NS} increased due to insufficient electrostatic control, while the SCEs were effectively suppressed at W_{NS} = 60 nm even for L_G below 100 nm. **d,e** I_D-V_D characteristics of n- and p-type devices with various L_Gs.

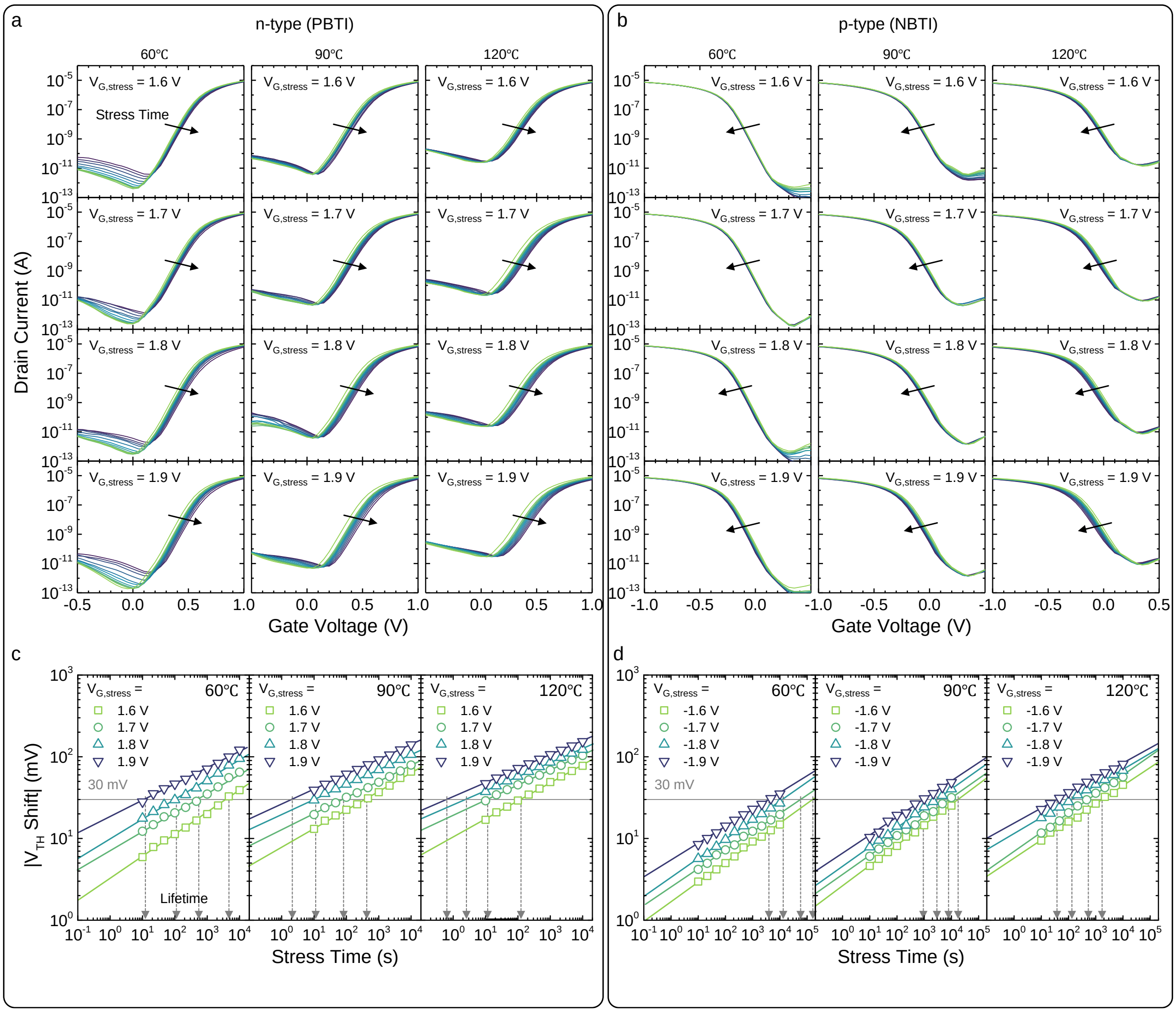

Supporting Information Figure S10. BTI extraction. a,b Transfer characteristics of fabricated n- and p-type NS GAA MPB-FETs under gate stress voltages ($V_{G,stress}$) ranging from ±1.6 to ±1.9 V were applied at various temperatures (60, 90, and 120°C) for evaluating BTI lifetime. **c,d** Resulting ΔV_{TH}s due to the bias temperature stress are plotted, where the V_{TH} was extracted using the constant current method ($I_{D,TH} = 10^{-7}$ A × (W_{NS}/L_G)). Here, the lifetime was defined based on a failure criterion of ΔV_{TH} = 30 mV.

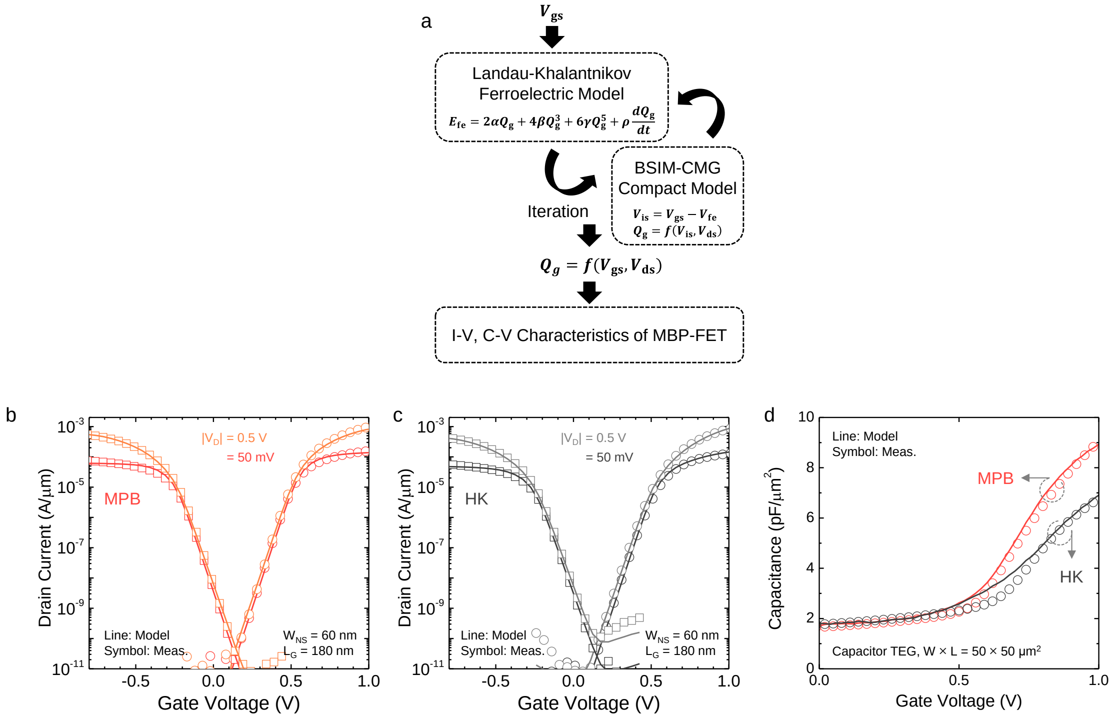

Supporting Information Figure S11. AC simulation of NS GAA MPB-FETs. a A flowchart of simulated BSIM CMG compact model with embedded L-K ferroelectric for circuit simulations. For a simulation of NS GAAFETs without a floating metal, the distributed charge model was employed, wherein the ferroelectric layer influences the local channel charge at each point along the channel. The BSIM-CMG compact model is self-consistently solved with Landau's FE model at various positions in the channel. The charge attained from this approach is then used for the calculation of the drain current and gate capacitance. (See **Supporting Information Note S4** for detailed compact modeling procedures.) **b-d** I_D-V_G and C_G-V_G curves of fabricated devices (MPB and HK) and compact model.

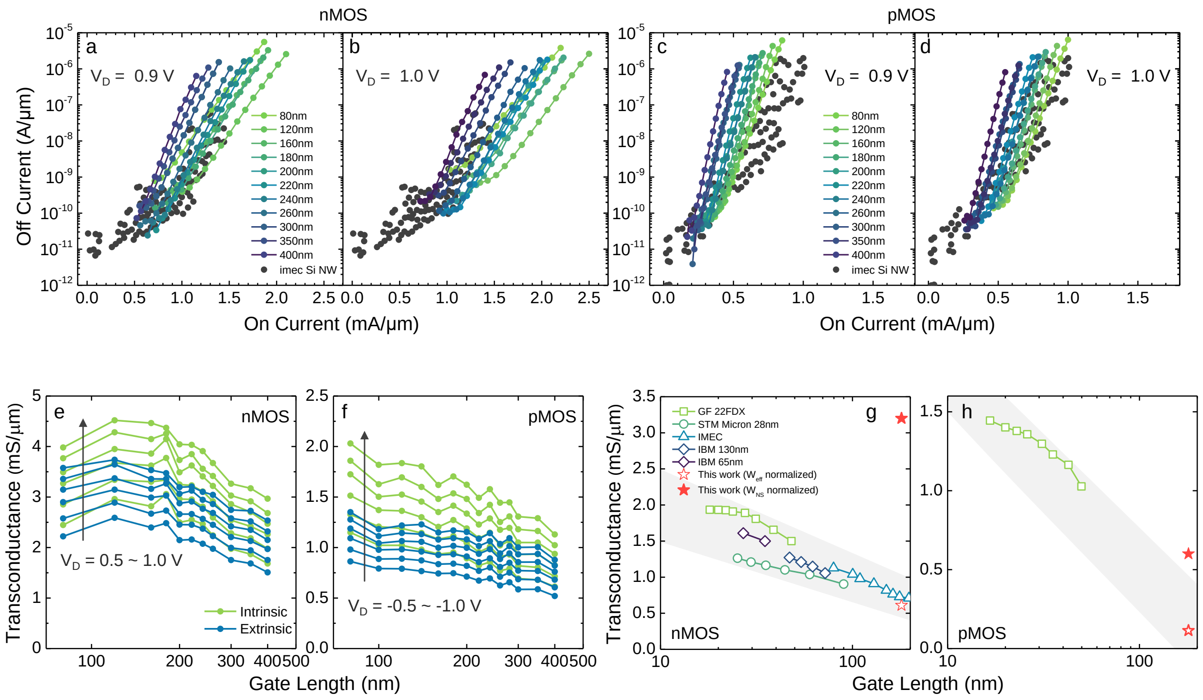

Supporting Information Figure S12. Benchmark of MPB-FETs with vertical stacked NW MOSFETs. a,b I_{ON}–I_{OFF} of n-type NS GAA MPB-FETs by channel length at V_D = 0.9 V and V_D = 1.0 V benchmarks compared to vertical stacked nanowire MOSFETs of IMEC. **c,d** Benchmark graphs of p-type NS GAA MPB-FETs. **e,f** g_m-L_G graphs of n-and p-type NS GAA MPB-FETs plotting the measured extrinsic g_m and the calculated intrinsic g_m. **g,h** g_m benchmarks compared to previously reported industrial logic devices.

Supporting Information Tables

Supporting Information Table S1. Benchmarking analysis against reported MPB films. The MPB thin film designed in this work shows a comparable peak dielectric constant (~45.5) to those reported in previous studies[2-5]. Particularly, through MFM capacitance measurements (**Figures S1d-f**) and the MFIS/FET level analyses (**Figures 1 and 2**), we demonstrated that the effective oxide thickness (EOT) can be scaled down to 0.257 nm, corresponding to a physical thickness of 3.0 nm, while maintaining the capacitance boosting effect.

	This Work	[2]	[3]	[4]	[5]
Zr content	75%	50% / 70%	70%	66%	75%
Peak dielectric constant	45.5	47 /43	38	49	53
Film thickness (nm)	6.0 / 3.0 (FET)	6.5 / 9.2	2.5~10	6.0	8
EOT (nm)	0.514 / 0.257	0.539 / 0.834	0.257~1.03	0.478	0.589

Supporting Information Table S2. Benchmarking analysis against reported ferroelectric-FETs including NCFETS with various topologies. The 3-decade average *SS* (SS_{3dec}) and footprint-normalized on-current (I_{ON}/W_{fp}) (at V_{DD} = 0.6 V) are compared. Since the transfer curves of each report exhibit different patterns, inferences were made for extracting *SS* and I_{on}, which is illustrated in Supporting Information Note S5 in detail. Our fabricated stacked GAA MPB-FETs firmly demonstrate superior performance for low-power logic devices, namely the high I_{on}/W_{fp} and near-thermionic *SS* without the hysteresis, owing to the GAA structure and reduced EOT by mixed-phase MPB HZO.

Ref.	Device type	Channel type	Material / thickness	Gate length (nm)	Footprint width (nm)	Hyster-esis	Sweep direction	I_{on}/W_{fp} @V_{DD}=0.6V (A/μm)	SS_{3dec} (mV/dec)
[6]	Planar (Si)	n-type	HZO / 5 nm	30,000	136,000	Yes	Forward<br>Reverse	3.00×10^{-6}<br>4.00×10^{-6}	68.8<br>47.1
[7]	Planar (Si)	n-type	HZO / 5 nm	2,000	20,000	Yes	Forward<br>Reverse	4.99×10^{-6}<br>8.24×10^{-6}	110<br>50
[8]	Fin (Si)	n-type	HZO / 5 nm	60	20	Yes	Forward<br>Reverse	1.04×10^{-4}<br>3.45×10^{-4}	93.7<br>58.6
		p-type					Forward<br>Reverse	1.12×10^{-4}<br>1.75×10^{-4}	65.7<br>65.7
[9]	Fin (Si)	n-type<br>p-type	HZO / 5 nm	30	30	No	-	8.61×10^{-5}<br>3.13×10^{-5}	82.8<br>92.8
[10]	Fin (Ge)	n-type<br>p-type	HZO / 7 nm	60<br>100	40<br>40	No<br>No	-<br>-	9.17×10^{-6}<br>2.39×10^{-5}	89.2<br>88.5
[11]	Planar (Si)	n-type	HZO / 5 nm	250	50,000	No	-	3.77×10^{-6}	61.7
[12]	Stacked NW (poly-Si)	n-type	HZO / 4.6 nm	250	16	No	-	2.39×10^{-5}	91.7
This work	Stacked NS (Si)	n-type<br>p-type	HZO / 3.0 nm	180	60	No	-	3.86×10^{-4}<br>1.92×10^{-4}	61.47<br>63.83

Supporting Information Notes

Supporting Information Note S1. XRD analysis

A high-resolution X-ray diffractometer (X' Pert Pro) was employed at an incident angle of 0.5°. Diffraction peaks from the stable monoclinic phase (m-phase, 28.5°) were almost absent, whereas diffraction peaks from the tetragonal (t-phase) and orthorhombic (o-phase) phases were observed. Because the diffraction peaks at the 2θ of 30.6° comprise a mixture of the orthorhombic 111 $(111)_o$ and tetragonal 011 $(011)_t$ phases, accurate separation of the two phases was challenging. Thus, for the deconvolution of the GIXRD spectra, the ratios were extracted under the assumption that $(111)_o$ and $(011)_t$ are located at 30.4° and 30.8°, respectively. [13, 14]

Supporting Information Note S2. Planar SOI FET process flow & R_{SD} experiment

The process flow for the planar SOI FETs (**Supporting Information Figure S3a**) is summarized in **Supporting Information Figure S3b**. Starting with a p-type SOI (100 nm) wafer, the active width (W_{ch}) ranging from 0.5 µm to 50 µm was patterned. Ultrawide actives were explicitly drawn to evaluate the FET-level quasi-static and small signal *C*-*V* characteristics, as well as to evaluate the fast I_D-V_G measurements. Subsequently, a non-self-aligned (NSA) source/drain (S/D) doping (As^+, 20 keV, of 2×10^{15} cm^{-2}) was performed. To control R_{SD}, the gate-to-S/D overlap length (L_{ov}) was varied across several groups on the same wafer, ranging from underlapping to overlapping configurations, as discussed in the following paragraph. Next, a gate stack composed of SiO_2, HZO (and HfO_2), and TiN was deposited using ALD and sputtering. Gates with lengths (L_G) ranging from 0.5 µm to 1 µm were then patterned, followed by PMA (500°C for 30 sec), back-end-of-line (BEOL), and HPA (18 Bar, 400°C, 1 h) processes.

A junction control experiment was conducted to verify that the observed I_{on} boosting in the mixed-phase HZO device was not merely a result of the reduced external resistance (R_{ext}). Differently designed S/D junctions resulted in variations in R_{ext}, as confirmed by the transfer characteristics shown in **Supporting Information Figure S3d**. The off-current, which is a combination of the GIDL and junction leakage current, was substantially lower at high V_D and negative V_G values in the underlapped device. Conversely, the drain current (I_D) increases under low V_D and high V_G conditions, where R_{ext} plays a dominant role in I_D. The on-resistance (R_{on}) versus 1/overdrive voltage (V_{ov}^{-1}) plot (**Supporting Information Figure S3e**) clearly demonstrates the differences in R_{ext} between the devices.

Supporting Information Note S3. Process flow of NS GAA MPB-FET

The process flow of the nanosheet GAA FETs is summarized in **Supporting Information Figure S8a**. The starting SOI wafer was then thinned to 35 nm. Multi-stacked epitaxial growth of SiGe/Si/SiGe/Si was conducted; single-crystalline Si epitaxial stacks were used as channel materials, whereas $Si_{0.75}Ge_{0.25}$ stacks were utilized as sacrificial layers for channel release. The active layer was formed via mixed-and-match lithography using photolithography/e-beam lithography, followed by inductively coupled plasma reactive ion etching (ICP-RIE) using HBr gas. The top-view scanning electron microscopy (SEM) images of the active layer, whose width (W_{NS}) ranged from 50 to 200 nm, are indicated in **Supporting Information Figure S8b**. Non-self-aligned source/drain (S/D) implantation (As^+, 20 keV, 2×10^{15} cm^{-2} for nSD and BF^{2+}, 20 keV, 2×10^{15} cm^{-2} for pSD) was introduced to lower the external resistance (i.e., contact and S/D resistance) after active formation. Before the channel-release procedure, blocking lithography was conducted to eliminate the gate-to-SD overlap resulting from the unintentionally etched SiGe region (i.e., the non-channel region). The gate-first process was used for convenient fabrication in a university fab rather than the replacement metal gate process, where the opening area for the channel release process was intrinsically self-aligned to the gate. SiGe layers were selectively wet-etched to suspend the nanosheet channel; the detailed procedure of selective etching is addressed later in this chapter. The NSA S/D dopant was activated by high temperature (900℃, 10 s) rapid thermal annealing (RTA). A gate stack composed of an IL (SiO_2) and an HK layer (HZO and HfO_2 for reference) was deposited using ALD, followed by TiN gate deposition, which was a combination of ALD TiN (to buffer sputter damage and fill the vertical gap between channels) and sputtered TiN for the gate electrode. Gate patterning was performed via mixed-and-match lithography using photolithography/e-beam lithography and ICP-RIE using Cl_2 gas. The L_Gs ranged from 50 to 200 nm, as shown in **Supporting Information Figure S8b**. A TiN wet-trimming process was implemented to lower the GIDL current induced by the remaining TiN spacer after gate-dry

etching. The subsequent self-aligned (SA) S/D ion implantation (P^+, 40 keV, 2×10^{15} cm^{-2} for nSD and BF^{2+}, 40 keV, 2×10^{15} cm^{-2} for pSD) and post-metallization annealing (PMA, 500℃, 30 s) for HZO crystallization were followed. The use of P^+ as the nSD dopant is advantageous because the dopant activation rate of a high dose at low temperatures is larger than that of As^+, and the lattice damage caused by ion implantation is mitigated. After the PMA process for HZO crystallization, the fabrication was completed through back-end-of-line (BEOL) processes, including interlayer dielectric (ILD) deposition using tetraethyl orthosilicate (TEOS), contact-hole etching, metal deposition, and pad patterning.

A key distinction between GAA and FinFET fabrication is the channel release process, which, despite its crucial importance, presents significant challenges in GAA manufacturing. The channel-release process for creating a single-crystalline Si nanosheet channel begins with repeated epitaxial growth of Si (as the channel material) and SiGe (as the sacrificial layer). After the active patterning, the sacrificial SiGe layer was selectively etched. In this study, wet etching using carboxylic acid (R–COOH) was selected because of its moderate etch rate (E/R) and high Si selectivity.[15] A mixture of 49% HF, 30% H_2O_2, and 99% CH_3COOH was prepared in a volume ratio of 2:160:50 to control the formation of peroxy acids (R-CO_3H), which were responsible for etching SiGe. It has been previously reported that the reaction rate of this solution is relatively slow, leading to variations in E/R with aging time.[16] However, the experimental results displayed that the E/R stabilized after approximately 96 h (four days) (**Supporting Information Figure S8e**). By measuring the Si E/R in the solution and comparing the Si thicknesses of the epi-grown and exposed regions in **Supporting Information Figure S8d**, a high etch selectivity of approximately 55:1 was confirmed, which remained consistent regardless of the aging duration. Utilizing the stabilized E/R, selective wet etching of SiGe was performed to attain a stable suspended Si NS channel, as supported by the cross-sectional SEM images in **Supporting Information Figure S8f**.

In **Supporting Information Figure S8i** (cross-sectional TEM image of the fabricated stacked NS GAA MPB-FET along the channel direction), it is noteworthy that the SiGe layers between the Si channels were over-etched in the source/drain (S/D) direction, leading to deeper penetration of the gate stack. Consequently, the L_G between the channels was longer than the designed L_G. This issue is not encountered when the epitaxial Si S/D technique, which is commonly employed in advanced industrial logic fabrication, is used.

Supporting Information Note S4. Compact modeling method for stacked NS GAA MPB-FET

For a simulation of NS GAAFETs without a floating metal, the distributed charge model was employed, wherein the ferroelectric layer modulates the local channel charge at each point along the transistor channel. The detailed simulation workflow is as follows.

First, the applied gate-to-source voltage (V_{gs}) undergoes a voltage division between the FE material and the NS GAAFET. Initially, this step involves calculating the total gate charge (Q_g) using the BSIM-CMG compact model. Based on Q_g, the electric field in the FE layer (E_{fe}) is determined based on the L-K model, which is grounded on the principle of charge conservation. This calculation links the polarization dynamics of the ferroelectric material to the local electric field within the capacitor. Then, the voltage across the ferroelectric layer (V_{fe}), derived from the L-K model, and the voltage across MOS capacitor (V_{is}) obtained from the BSIM-CMG compact model, are iteratively checked to ensure consistency with the total applied V_{gs}. This step employs an iterative solver to adjust Q_g, V_{fe}, and V_{is} until convergence is achieved, ensuring accurate modeling of the coupled system. Once convergence is reached, the finalized gate charge is used to compute the transistor's drain current and gate capacitance. These parameters are then utilized to extract the I_D-V_G and C-V characteristics of the MPB-FET.

Supporting Information Note S5. Benchmarking methods

When benchmarked against the transconductance values from industry RF technologies, the measured transconductance ($g_{\mathrm{m}} = \partial I_{\mathrm{D}}/\partial V_{\mathrm{G}}$) and output conductance ($g_{\mathrm{DS}} = \partial I_{\mathrm{D}}/\partial V_{\mathrm{D}}$) are affected by the source/drain series resistance (R_{S} and R_{D}), as they reduce the voltage drop across the channel region. In particular,

$$V_{\mathrm{GS}} - I_{\mathrm{D}} R_{\mathrm{S}}$$
$$V_{\mathrm{DS},i} = V_{\mathrm{DS}} - I_{\mathrm{D}}(R_{\mathrm{S}} + R_{D}),$$

, where $V_{\mathrm{GS,i}}$ and $V_{\mathrm{DS,i}}$ are the gate-to-source and drain-to-source voltages intrinsic to the channel, respectively. The parasitic R_{S} and R_{D} can be extracted from the y-intercept (R_{P}) of R_{SD} ($R_{\mathrm{S}} + R_{\mathrm{D}}$) – $1/V_{\mathrm{OD}}$, where $V_{\mathrm{OD}} = V_{\mathrm{GS}} - V_{\mathrm{TH}}$, as for large V_{OD}, the channel resistance is inversely proportional to V_{OD}, and $R_{\mathrm{S}} = R_{\mathrm{D}} = R_{\mathrm{P}}/2$ as the device is symmetric. Consequently, the intrinsic transconductance ($g_{\mathrm{m,i}}$) attained by bedding the parasitic series resistance can be expressed as follows:

$$g_{\mathrm{m},i} = g_{\mathrm{m}}[1 - g_{\mathrm{m}} R_{\mathrm{D}} - g_{\mathrm{DS}}(R_{\mathrm{S}} + R_{\mathrm{D}})]^{-1}.$$

In **Supporting Information Figures S12e,f** presents the extrinsic and intrinsic transconductance characteristics for n-type and p-type NS GAA MPB-FETs with varying L_{G}s, measured across different V_{D}s. The $g_{\mathrm{m,i}}$ is benchmarked in **Supporting Information Figures S10g,h** against previous studies on n-type and p-type MOSFETs featuring HfO_2-based conventional high-k (HK) gate stacks.[17, 18] Utilizing the mixed-phase HZO gate stack, the $g_{\mathrm{m,i}}$ demonstrated similar L_{G} scaling trends when normalized by the effective width (W_{eff}) and exceeded the overall $g_{\mathrm{m,i}}$-L_{G} trend associated with conventional HK gate stacks when normalized by the footprint (i.e., W_{NS}). This enhancement in transconductance is attributed to the improved capacitance of the mixed-phase HZO gate oxide, which did not compromise electron or hole transport efficiency.

Supporting Information Note S6. Effective mobility extraction of stacked NS GAA FETs

In order to accurately extract the effective mobility (μ_{eff}) of the HK and MPB devices fabricated on the stacked NS GAA platform, the removal of the extrinsic component, R_P, was preceded by following manner.

From the transfer characteristics of the NS GAA CMOS (**Figure 3b**), the on-resistance (R_{on}) can be expressed as:

$$R_{\mathrm{on}} = R_{\mathrm{ch}} + R_P = V_{\mathrm{D}}/I_{\mathrm{D}}(V_{\mathrm{G}} = \pm 1\ \mathrm{V})$$

, where the R_P is the parasitic (external) resistance, and the R_{ch} is the channel resistance, which is function of V_G or $V_{OD} = V_G - V_{TH}$. As described in Supporting Information Note S4, as for large V_{OD}, R_{ch} is linearly proportional to $1/V_{OD}$. Therefore, by plotting R_{on} vs. V_{OD}, the y-intercept of the trendline gives an estimate of R_P.

By eliminating R_P from R_{on}, the intrinsic R_{ch} is obtained, and the μ_{eff} in the inversion region ($V_G = \pm 1$ V) is calculated using the following equation:

$$\mu_{\mathrm{eff}} = \frac{L}{W R_{\mathrm{ch}} Q_{\mathrm{inv}}}\ (V_G = \pm 1\ \mathrm{V})$$

Here, the inversion charge (Q_{inv}) can be calculated by integrating gate capacitance (C_{gg}) from the measured *C-V* curve (**Figure 1e**) as:

$$Q_{\mathrm{inv}} = \frac{1}{A}\int_{V_{\mathrm{fb}}}^{V_{\mathrm{inv}}} C_{gg}(v_{\mathrm{g}})\,dv_{\mathrm{g}}$$

, where the inversion V_G (V_{inv} = 1) V.

Supporting Information Note S7. Performance comparison metrics

Since the discovery of ferroelectricity in CMOS-compatible doped HfO, extensive research has been conducted on HfSiO (HSO)- or HZO-based ferroelectric-FET including NCFETs with various topologies[6-12]. Multiple reports have demonstrated an *SS* of sub-60 mV/dec based on the minimum point *SS*[7-11] or exhibited a steep slope with ferroelectric counter-clockwise hysteresis[6-8], implying a transient NC driven by polarization switching during a slow DC sweep. Additionally, numerous studies have merely validated the effect of NC HZO on 2D planar-structured transistors[6, 7], demonstrating poor current drivability, and did not verify its applicability to advanced logic structures.

To ensure a fair comparison across different transfer curves, we employed the following methodology.

1) Footprint-normalized on-current: Because the transfer curves of each report had varying V_{TH} values and were measured under different V_D conditions, it was difficult to compare the drivability across the reported data fairly. Here, the I_{on} represents the I_D extracted under the conditions of V_G = 0.6 V when I_{off} = 10^{-10} A/μm and V_D = 0.6 V. The I_{on} at V_D = 0.6 V was evaluated under the simple assumption that the MOSFET is in the linear region before reaching the saturation V_D ($V_{D,sat}$). Additionally, the I_{on}s were compared with the footprint-normalized value I_{on}/W_{fp}, where W_{fp} refers to the drawn channel width. This comparison emphasizes the current drivability of devices in the same area, which has greater significance in 3D structures.

2) Subthreshold swing: To prevent overestimation of *SS* in a limited range, the *SS* was extracted equitably as the average value over a 3-decade range of the normalized I_D (I_D/W_{fp}), specifically between 10^{-10} and 10^{-7} A/μm.

The exceptional performance of the NS GAA MPB-FETs demonstrated in this study suggests a strong direction of future logic technologies. It is strongly anticipated that further enhancements in the current characteristics of NS GAA MPB-FETs can be achieved through the application of advanced logic techniques, including contact silicidation, S/D junction

engineering (epitaxy S/D), replacement metal gate (gate-last) processes, and by shortening the L_G.

Supporting Information References

[1] K. Lee, J. Lee, S. Kim, R. Lee, S. Kim, M. Kim, J. H. Lee, S. Kim, B. G. Park, *IEEE Trans. Nanotechnol.* **2020**, *19*, 168.

[2] M. H. Park, Y. H. Lee, H. J. Kim, Y. J. Kim, T. Moon, K. D. Kim, S. D. Hyun, C. S. Hwang, *ACS Applied Materials & Interfaces* **2018**, *10*, 42666.

[3] K. Ni, A. Saha, W. Chakraborty, H. Ye, B. Grisafe, J. Smith, *IEEE Int. Electron Devices Meet.* **2019**.

[4] D. Das, B. Buyantogtokh, V. Gaddam, S. Jeon, *IEEE Tran. on Electron Device* **2022**, *69*, 103.

[5] S. Kim, S. H. Lee, M. J. Kim, W. S. Hwang, H. S. Jin, B. J. Cho, *IEEE Electron Device Lett.* **2021**, *42*. 517.

[6] M. H. Lee, P. G. Chen, C. Liu, K. Y. Chu, C. C. Cheng, M. J. Xie, S. N. Liu, J. W. Lee, S. J. Huang, M. H. Liao, M. Tang, K. S. Li, M. C. Chen, *IEEE Int. Electron Devices Meet. IEDM* **2015**.

[7] P. Sharma, K. Tapily, A. K. Saha, J. Zhang, A. Shaughnessy, A. Aziz, G. L. Snider, S. Gupta, R. D. Clark, S. Datta, *IEEE Symp. VLSI Technol.* **2017**.

[8] K. S. Li, Y. J. Wei, Y. J. Chen, W. C. Chiu, H. C. Chen, M. H. Lee, Y. F. Chiu, F. K. Hsueh, B. W. Wu, P. G. Chen, T. Y. Lai, C. C. Chen, J. M. Shieh, W. K. Yeh, S. Salahuddin, C. Hu, *IEEE Int. Electron Devices Meet. IEDM* **2018**.

[9] K. S. Li, P. G. Chen, T. Y. Lai, C. H. Lin, C. C. Cheng, C. C. Chen, Y. J. Wei, Y. F. Hou, M. H. Liao, M. H. Lee, M. C. Chen, J. M. Sheih, W. K. Yeh, F. L. Yang, S. Salahuddin, C. Hu, *IEEE Int. Electron Devices Meet. IEDM* **2015**.

[10] C. J. Su, Y. T. Tang, Y. C. Tsou, P. J. Sung, F. J. Hou, C. J. Wang, S. T. Chung, C. Y. Hsieh, Y. S. Yeh, F. K. Hsueh, K. H. Kao, S. S. Chuang, C. T. Wu, T. Y. You, Y. L. Jian, T. H. Chou, Y. L. Shen, B. Y. Chen, G. L. Luo, T. C. Hong, K. P. Huang, M. C.

Chen, Y. J. Lee, T. S. Chao, T. Y. Tseng, W. F. Wu, G. W. Huang, J. M. Shieh, W. K. Yeh, Y. H. Wang, *IEEE Symp. VLSI Technol.* **2017**.

[11] D. Kwon, K. Chatterjee, A. J. Tan, A. K. Yadav, H. Zhou, A. B. Sachid, R. Dos Reis, C. Hu, S. Salahuddin, *IEEE Electron Device Lett.* **2018**, *39*, 300.

[12] S. Y. Lee, C. C. Lee, Y. S. Kuo, S. W. Li, T. S. Chao, *IEEE J. Electron Devices Soc.* **2021**, *9*, 236.

[13] J. Müller, T. S. Böscke, U. Schröder, S. Mueller, D. Bräuhaus, U. Böttger, L. Frey, T. Mikolajick, *Nano Lett.* **2012**, *12*, 4318.

[14] M. H. Park, H. Joon Kim, Y. Jin Kim, W. Lee, T. Moon, C. Seong Hwang, *Appl. Phys. Lett.* **2013**, *102*, 242905.

[15] D. -I. Bae and B. -D. Choi, *Electron. Lett.* **2020**, *56*, 400.

[16] Y. H. Kil, J. H. Yang, S. Kang, T. S. Jeong, T. S. Kim, K. H. Shim, *J. Semicond. Technol. Sci.* **2013**, *13*, 668.

[17] J. Jo, C. Shin, *Electron. Lett.* **2015**, *51*, 106.

[18] Y. G. Xiao, M. H. Tang, J. C. Li, C. P. Cheng, B. Jiang, H. Q. Cai, Z. H. Tang, X. S. Lv, X. C. Gu, *Appl. Phys. Lett.* **2012**, *100*, 083508.
